# Recombination in *pe/ppe* genes contributes to genetic variation in *Mycobacterium tuberculosis* lineages

**DOI:** 10.1186/s12864-016-2467-y

**Published:** 2016-02-29

**Authors:** Jody E. Phelan, Francesc Coll, Indra Bergval, Richard M. Anthony, Rob Warren, Samantha L. Sampson, Nicolaas C. Gey van Pittius, Judith R. Glynn, Amelia C. Crampin, Adriana Alves, Theolis Barbosa Bessa, Susana Campino, Keertan Dheda, Louis Grandjean, Rumina Hasan, Zahra Hasan, Anabela Miranda, David Moore, Stefan Panaiotov, Joao Perdigao, Isabel Portugal, Patricia Sheen, Erivelton de Oliveira Sousa, Elizabeth M. Streicher, Paul D. van Helden, Miguel Viveiros, Martin L. Hibberd, Arnab Pain, Ruth McNerney, Taane G. Clark

**Affiliations:** Department of Pathogen Molecular Biology, Faculty of Infectious and Tropical Diseases, London School of Hygiene & Tropical Medicine, Keppel Street, WC1E 7HT London, UK; KIT Biomedical Research, Royal Tropical Institute, Amsterdam, Netherlands; Department of Science and Technology and National Research Foundation Centre of Excellence for Biomedical Tuberculosis Research, and Medical Research Council Centre for Molecular and Cellular Biology, Division of Molecular Biology and Human Genetics, Faculty of Medicine and Health Sciences, Stellenbosch University, Tygerberg, South Africa; Faculty of Epidemiology and Population Health, London School of Hygiene & Tropical Medicine, WC1E 7HT London, UK; Karonga Prevention Study, Lilongwe, Malawi; National Mycobacterium Reference Laboratory, Porto, Portugal; Centro de Pesquisas Goncalo Moniz, Fundacao Oswaldo Cruz Bahia R, Salvador, Bahia Brazil; Department of Medicine, Lung Infection and Immunity Unit, Division of Pulmonology & UCT Lung Institute, University of Cape Town, Cape Town, Western Cape South Africa; Institute of Infectious Diseases and Molecular Medicine, University of Cape Town, Cape Town, Western Cape South Africa; Laboratorio de Enfermedades Infecciosas, Laboratorios de Investigación y Desarrollo, Facultad de Ciencias y Filosofía, Universidad Peruana Cayetano Heredia, Lima, Peru; Department of Pathology and Laboratory Medicine, The Aga Khan University, Stadium Road, Karachi, Pakistan; National Center of Infectious and Parasitic Diseases, 1504 Sofia, Bulgaria; Universidade de Lisboa, Lisbon, Portugal; Grupo de Micobactérias, Unidade de Microbiologia Médica, Global Health and Tropical Medicine (GHTM), Instituto de Higiene e Medicina Tropical, Universidade NOVA de Lisboa (IHMT/UNL), Lisbon, Portugal; Biological and Environmental Sciences and Engineering Division, King Abdullah University of Science and Technology, Thuwal, Kingdom of Saudi Arabia

## Abstract

**Background:**

Approximately 10 % of the *Mycobacterium tuberculosis* genome is made up of two families of genes that are poorly characterized due to their high GC content and highly repetitive nature. The PE and PPE families are typified by their highly conserved N-terminal domains that incorporate proline-glutamate (PE) and proline-proline-glutamate (PPE) signature motifs. They are hypothesised to be important virulence factors involved with host-pathogen interactions, but their high genetic variability and complexity of analysis means they are typically disregarded in genome studies.

**Results:**

To elucidate the structure of these genes, 518 genomes from a diverse international collection of clinical isolates were *de novo* assembled. A further 21 reference *M. tuberculosis* complex genomes and long read sequence data were used to validate the approach. SNP analysis revealed that variation in the majority of the 168 *pe/ppe* genes studied was consistent with lineage. Several recombination hotspots were identified, notably *pe_pgrs3* and *pe_pgrs17*. Evidence of positive selection was revealed in 65 *pe/ppe* genes, including epitopes potentially binding to major histocompatibility complex molecules.

**Conclusions:**

This, the first comprehensive study of the *pe* and *ppe* genes, provides important insight into *M. tuberculosis* diversity and has significant implications for vaccine development.

**Electronic supplementary material:**

The online version of this article (doi:10.1186/s12864-016-2467-y) contains supplementary material, which is available to authorized users.

## Background

Tuberculosis disease (TB) is a major global public health problem, with control becoming difficult due to increasing drug resistance and in some populations HIV co-infection [1]. The available vaccine, Bacillus Calmette–Guérin (BCG), has limited efficacy and recent attempts to develop more effective protective vaccines have not been successful [2]. TB is caused by bacteria of the *Mycobacterium tuberculosis* complex, which have low overall genetic diversity and a striking clonal population structure. *M. tuberculosis sensu stricto* consists of seven lineages, including four that are predominant; 1 Indo-Oceanic, 2 East-Asian including Beijing, 3 East-African-Indian, 4 Euro-American [3]. These lineages are postulated to have differential impacts on pathogenesis, disease outcome and vaccine efficacy [4–7]. For example, modern lineages, such as Beijing and Euro-American Haarlem strains exhibit more virulent phenotypes compared to ancient lineages, such as East African Indian^8^. Whilst some genetic differences between lineages have been identified^3^, the molecular mechanisms responsible for differences in pathogenesis and virulence remain largely unknown [8].

Two groups of proteins, the PE and PPE families have been implicated in immune evasion and virulence [9]. Members of the *pe/ppe* gene families are characterized by the presence of proline-glutamate (PE) and proline-proline-glutamate (PPE) signature motifs near the N-terminus of their gene products [10]. The *pe* (99 loci) and *ppe* (69) gene families constitute ~7–10 % of the coding potential of *M. tuberculosis* and are scattered throughout the genome [9]. The families can be subdivided based on similarities in their N-terminal regions [11]. Many of the *pe* and *ppe* gene products are predicted to be localised to the cell membrane or secreted including those in the PE_PGRS domain containing subgroup and the PPE_MPTR domain containing subgroup [12, 13]. It has been speculated that these proteins may play a role in virulence [14]. *Pe/ppe* genes are differentially expressed during infection [15] and some PE/PPE proteins have been shown to elicit immune responses by the host [14, 16] and there is evidence that the PGRS domain can inhibit antigen processing [16, 17].

Whilst *pe_pgrs* and *ppe_mptr* genes represent some of the most variable *M. tuberculosis* regions, some members of the *pe/ppe* family are conserved across strains and species, therefore implying different functional roles. Only the protein structures of PE25 and PPE41 have been characterised [18], and in lieu of experimental and functional work, insights into their function and interaction partners must come from in silico analysis of large-scale ‘omics data. However, due to the repetitive nature and high GC content genetic variation in the *pe/ppe* genes, it has been difficult to characterize them using traditional mapping approaches, leading to their systematic exclusion from analysis [18]. There have been conflicting studies reporting either high or little or no sequence divergence [19–21], but studies have been limited by the number of genes and diversity of strains analysed.

There is a need to fully characterize *pe/ppe* family sequence diversity across strain-types to provide better understanding of these genes and their possible role in virulence and immune evasion. The availability of high throughput short sequencing technologies has revolutionized the study of *M. tuberculosis* genetic diversity. In an attempt to characterize these elusive genes we have performed whole genome assembly on next generation sequence data with a high depth of coverage across the *pe/ppe* gene regions from 518 clinical and experimental isolates. These isolates represent the four major lineages, each with known informative barcoding SNPs [3]. The approach was validated by examination of 21 reference genomes from established databases (www.tbdb.org; www.ebi.ac.uk), including 2 new strains with complete genomes sequenced using long read Pacific Bioscience (PacBio) technology [22, 23].

## Results

### Assembly of *M. tuberculosis* genomes

Conventional alignment-based analysis approaches have been of limited use in analysis of highly repetitive loci, including the *pe/ppe* genes. Here, we *de novo* assembled the genomes of 518 samples from 9 different countries covering the four main lineages (1 (n = 42), 2 (n = 38), 3 (n = 53), and 4 (n = 385)), with high sequence coverage in *pe/ppe* genes (mean 233-fold, range 100–1544) (Additional file 1: Tables S1 and S2). For each sample, at least 120 of the 168 *pe/ppe* genes were fully assembled and at least 90 % assembled for the remaining 48 genes (Additional file 1: Table S3). This level of assembly quality ensured low levels of assembly fragmentation and minimised poor gene characterization. Subsequent analysis involving manual inspection or re-mapping of reads to the assemblies using REAPR software, revealed all genes (168 *pe/ppe*; 3,654 other genes; 2,820 with an assigned function) to be of high quality (median REAPR score of 1 across all bases, reflecting high levels of accuracy in genome assemblies). A further 21 independent complete reference genomes representing all four lineages (Additional file 1: Table S1), were aligned against H37Rv to call variants, and used to further validate the results found in the assembled dataset.

#### Variant detection and population genetic analysis

A total of 50,539 genome-wide SNPs were identified by comparing the 518 assembled genomes to the H37Rv (lineage 4, Euro-American T) reference strain. Of these, 5,853 (11.6 %) SNPs were located within *pe/ppe* regions, with greater density than the rest of the genome (median SNPs per kb: *ppe/pe* = 12.9, non-*ppe/pe* = 9.1, Wilcoxon *P* < 2.2 × 10^−14^). In the 257 Malawi samples, our assembly procedure revealed 3,467 additional SNP variants genome-wide (1,438 (41.5 %) SNPs in 72 *pe/ppe* genes) compared to the standard approach of aligning short reads to the H3Rv reference. Of the 50,539 SNPs inferred from the assemblies, the majority (45,681, 90.3 %) were located in coding regions from all genes and consisted of 28,235 (61.8 %) non-synonymous SNPs and 17,446 (38.2 %) synonymous SNPs. This observation is in agreement with the higher abundance of non-synonymous mutations reported in the literature [19]. A large number of rare variants (i.e. present in only one isolate) were observed in all lineages, indicative of purifying selection and population expansion described by others [24]. The peaks in the spectrum represent a number of SNPs that are fixed in all isolates from sub-lineages (Additional file 2: Figure S1).

**Fig. 1:**
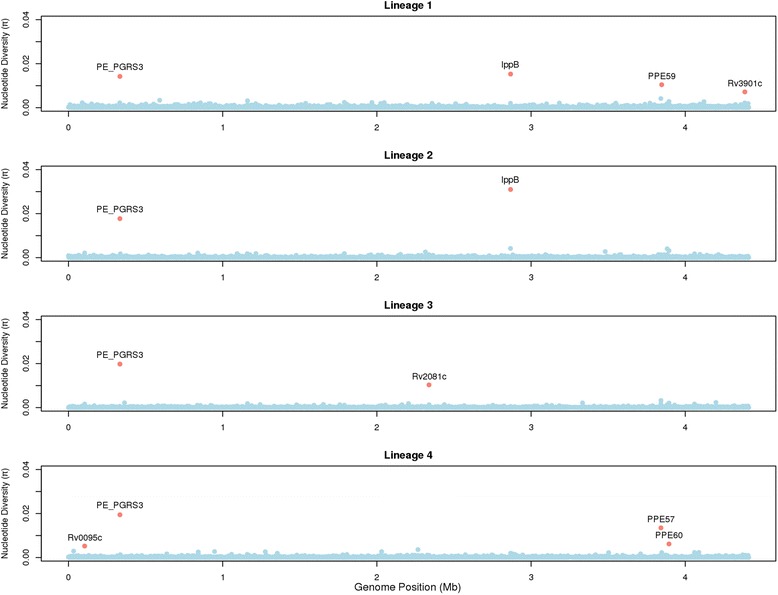
Nucleotide diversity (*π*) across the genome by lineage; genes with high diversity (*π* > 0.003) are highlighted. The *pe_pgrs3* gene appears to have high nucleotide diversity in all lineages. Some lineage-specific hotspots are seen in lineage 1 (*ppe59* and *Rv3901c*), lineage 3 (R*v2081c*) and lineage 4 (*ppe57* and *ppe60*)

The ratio of non-synonymous to synonymous mutations was similar in *pe/ppe* and other genes (median: *pe/ppe* genes = 1.65, other genes = 1.75, Wilcoxon *P* = 0.68). The density of non-synonymous mutations was 2.98 times greater in *pe/ppe* genes compared to others (*pe/ppe* genes: 1 every 3933 bp, other genes: 1 every 11,706 bp, Wilcoxon *P* < 0.0001), consistent with another report [25]. When analysed by sub-family we observed the greatest ratio of densities in the *pe_pgrs* genes (*pe_pgrs* 3.89) compared to the other types (*ppe* 1.75, *pe* (non-*pe_pgrs*) 1.80), similar to that reported previously [25]. The nucleotide diversity (π) was ~2-fold greater in the *pe/ppe* genes (median: *pe/ppe* genes 2.7 × 10^−4^, other genes 1.4 × 10^−4^, Wilcoxon *P* < 1.4 × 10^−10^). Although estimates of genetic diversity may be influenced by sampling bias, nucleotide diversity varied by lineage, being greater in lineage 1 (Indo-Oceanic median: *pe/ppe* 1.7 × 10^−4^, other 9.0 × 10^−5^) and lower in lineage 2 (East-Asian median: *pe/ppe* 7.3 × 10^−5^, other 0) (Additional file 1: Table S2), all consistent with previous work [3]. Loci identified as being highly diverse (π > 0.003, top 0.2 %, Table 1, Fig. 1), included 5 *pe/ppe* genes (*pe_pgrs3, pe_pgrs4, ppe57*, *ppe59* and *ppe60*), and 3 others *(Rv0030*, *Rv0095c and lppB*). The diversity per gene was compared to those from 21 complete reference genomes, and peaks were observed at *Rv0095c, pe_pgrs3*, *pe_pgrs4, ppe57 and ppe60,* independently supporting five out of the eight loci identified in the 518 global samples (Additional file 3: Figure S2).

**Table 1:** Loci that are highly diverse, with recombination, or under selective pressure

Gene	Locus	No. SNPs	Diversity *π*	*phi p-value*	*phi p*-value^a^	*dN/dS (w)*	No. sites^b^	Lineage specific *phi*
*Rv0030*	*Rv0030*	3	**0.0033**	1.000	1.000	-	0	-
*Rv0095c*	*Rv0095c*	10	**0.0059**	**0.005**	**0.021**	10.13	3	-
*Rv0182c*	*sigG*	3	0.0003	**0.046**	**0.046**	-	0	-
*Rv0278c*	*pe_pgrs3*	130	**0.0193**	**<0.001**	**<0.001**	10.5	49	1,3,4
*Rv0279c*	*pe_pgrs4*	49	**0.0035**	**0.001**	0.419	10.5	20	-
*Rv0282*	*eccA3*	5	0.0005	**0.007**	0.210	9.697	6	-
*Rv0850*	*Rv0850*	2	**0.0031**	1.000	1.000	9.264	4	-
*Rv0978c*	*pe_pgrs17*	9	0.0005	**0.003**	1.000	10.495	9	-
*Rv1148c*	*Rv1148c*	18	0.0022	**<0.001**	0.015	10.492	5	4
*Rv1793*	*esxN*	6	0.0023	**0.034**	0.159	9.694	2	4
*Rv1945*	*Rv1945*	18	0.0010	**<0.001**	**0.026**	10.433	5	-
*Rv2048c*	*pks12*	80	0.0008	**<0.001**	**0.012**	10.5	79	4
*Rv2543*	*lppA*	8	0.0015	**0.006**	**0.002**	10.036	5	4
*Rv2544*	*lppB*	60	**0.0123**	**<0.001**	**<0.001**	5.336	33	1,2,4
*Rv3425*	*ppe57*	31	**0.0154**	0.431	1.000	10.5	21	-
*Rv3429*	*ppe59*	19	**0.0041**	**<0.001**	0.084	10.419	29	4
*Rv3466*	*Rv3466*	6	0.0010	**0.004**	0.373	7.757	3	-
*Rv3478*	*ppe60*	105	**0.0061**	**<0.001**	**0.004**	7.502	54	4
*Rv3619c*	*esxV*	3	0.0022	**0.025**	1.000	10.391	2	-

*π* nucleotide diversity, *phi* recombination, *NS* not significant

^a^after removing sites under selection

^b^number of sites under selection using the Bayes Empirical Bayes method

Bolded refers to π > 0.003 *or phi p*-value < 0.05

### Phylogenetics

**Fig. 2:**
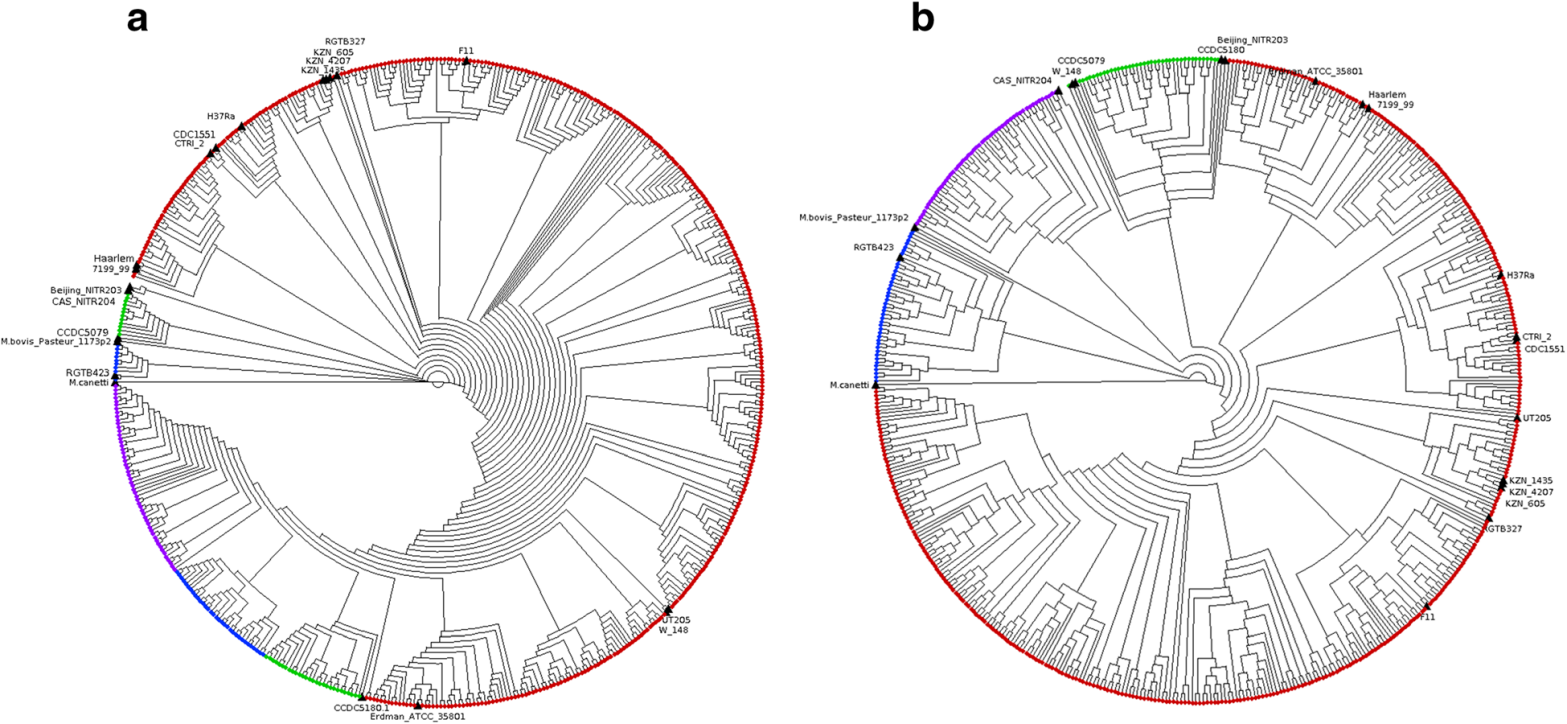
Phylogenetic tree constructed with SNPs. **a** *pe/ppe* genes (5,404 SNPs, 10 % of the genome); the *pe_pgrs3* gene was identified as having SNPs leading to the lineages not perfectly clustering, potential evidence of recombination affecting these loci. **b** *pe/ppe* genes excluding *pe_pgrs3* (5,572 SNPs, ~10 % of the genome). Clear clustering according to lineage can be seen (Lineage 1 (Indo-Oceanic, *green*), lineage 2 (East-Asian (Beijing), *blue*), lineage 3 (East-African-Indian, *purple*), lineage 4 (Euro-American, *red*)). Reference genomes are labelled. *M. canetti* is annotated in cyan

To examine the link between genetic variation and lineage, a phylogenetic tree was constructed using the 50,539 SNPs. It revealed clustering by lineage, thereby further validating the quality of the assembled genomes (Additional file 4: Figure S3). However, a similar analysis using 5,853 *pe/ppe* specific SNP positions led to a tree with lineage 2 being split into two distinct clades, surrounded by lineage 4 strains (Fig. 2a). Subsequent analysis using SNP-based population differentiation *F*_*ST*_ and site-specific log likelihood scores approaches (Additional file 5: Figure S4) revealed that the *pe_pgrs3* gene (genomic position 333 kb, lineage 2 – 104 SNPs differentiating) was predominantly responsible for the ambiguity. Removal of the 281 SNPs in the *pe_pgrs3* gene led to a *pe/ppe*-based tree that clustered by lineage (Fig. 2b), very similar in topology to that based on the genome-wide SNPs (Additional file 4: Figure S3). This demonstrated that a core set of *pe/ppe* SNPs appears to be lineage specific, and further analysis revealed a set of 87 (1.4 %) SNPs (66 non-synonymous) that were lineage specific, potentially forming the basis of a lineage-specific molecular barcode (Additional file 1: Table S4). None of these 87 mutations were present in *M. bovis* (GCA_000195835) or *M. africanum* (NC_015758.1) sequences, and therefore robust as *M. tuberculosis* lineage-specific markers. Using only the *pe_pgrs3* SNPs led to a tree with two large clades (Additional file 6: Figure S5), one containing H37Rv and strains with similar sequence, and the other consistent with isolates similar to M323 and 18b strains (Additional file 1: Table S1b), both undergoing recent sequencing using PacBio long read technology. The M323, 18b and similarly clustered assembled samples have a *pe_pgrs3* gene with conserved regions at both 3′ and 5′ ends, surrounding a highly similar hypervariable core*.* A different hypervariable core is present in H37Rv and similarly clustered assemblies, which interestingly is also present in the *pe_pgrs4* gene of 18b, and recombination is a potential explanation.

#### Recombination detection

**Fig. 3:**
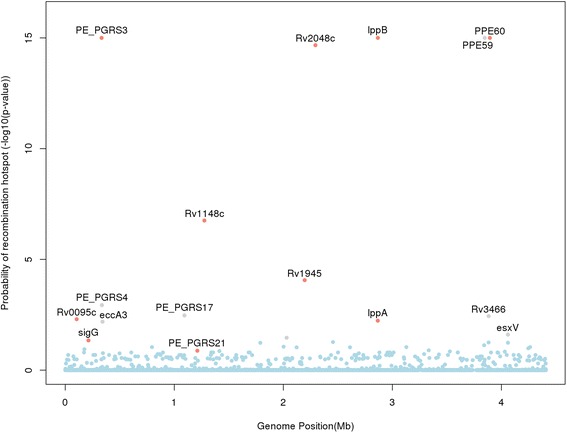
Evidence of recombination at a gene level. A Manhattan plot showing genes that are likely to be recombination hotspots. The (−log10) *p*-value for recombination is plotted against genome position. All genes with *p*-values <0.05 are labelled. Genes labelled in colour grey *(eccA3, pe_pgrs4, pe_pgrs17, ppe59, Rv3466 and esxV)* become statistically non-significant after removing sites under selection

Although it has been thought that *M. tuberculosis* undergoes little or no homologous recombination, PE_PGRS and PPE_MPTR families contain long domains comprised of series of tandem repeats, giving them a higher propensity to undergo recombination. There is published evidence of intra-chromosomal cross-over ahead of a few loci [9], including *pe_pgrs3, pe_pgrs4*, and *ppe1* [26]. We hypothesized that recombination may be the reason for the observed high genetic diversity and distortion in the *pe/ppe* tree. We applied the pairwise homoplasy index (*phi*) method [27] genome-wide to establish if there was any evidence of recombination in *pe_pgrs3* and other loci (Fig. 3). The method calculates a *p*-value (*phi* P) of observing the sequence data under the null hypothesis of no recombination. The analysis revealed 16 genes with potential recombination events (*phiP* < 0.05) present across all lineages: 5 in *pe/ppe* genes (*pe_pgrs3, pe_pgrs4, pe_pgrs17*, *ppe59 and ppe60*), and 11 others (*Rv0095, sigG, eccA3, Rv1148, esxN, Rv1945, pks12, lppA, lppB, Rv3466* and *esxV*).

It could be expected that the vast majority of any genomic recombination events are intra-lineage and that these events will pass unnoticed by other analyses, especially in studies of small sample size. Lineage-specific hotspots were also present (Additional file 7: Figure S6), including possible pathogenicity factors *lppA/lppB* in lineage 2 (Beijing) and *pe_pgrs3* in lineage 4. An analysis of the 21 complete reference genomes revealed an overall high degree of concordance of the homoplasy *phi* statistic with the assembled data, with six recombination peaks in common (*Rv0095c, pe_pgrs3, pe_pgrs4, pe_pgrs17*, *Rv1148c* and *Rv1945*) (Additional file 8: Figure S7). Together, these results provide evidence for recombination.

#### Detecting selection pressure

It is possible that recombination and population expansion [24] could have introduced not only the observed increased diversity in the *pe/ppe* genes, but contributed to an excess of non-synonymous mutation diversity in general; especially in genes expected to be under positive or diversifying selection such as the cell wall component genes [24]. Proteins in contact with the host proteome could be under pressure to change their amino acid sequence in order to avoid detection or unfavourable interaction with the host immune system. We decided to investigate the role of selection in the *pe/ppe* genes compared to other categories of genes. The distribution of *dN/dS* values (denoted *ω,* = 1 neutral evolution, >1 positive selection, <1 purifying selection), calculated for each gene across all sites and branches of the phylogenetic tree, was similar between *pe/ppe* and other genes (median *ω*: *pe/ppe* genes 0.81, other genes 0.73; Wilcoxon *P* = 0.16). These values are broadly similar to those previously reported on much lower numbers of samples and *pe/ppe* genes [25]. The genes were further divided into functional Clusters of Orthologous Groups (COG) categories [28]. Higher median *ω* values were observed in genes associated with signal transduction mechanisms (median = 0.95), perhaps due to their contact with the host, and the lowest values found in genes associated with RNA processing and modification (median = 0.38) (Additional file 9: Figure S8).

**Fig. 4:**
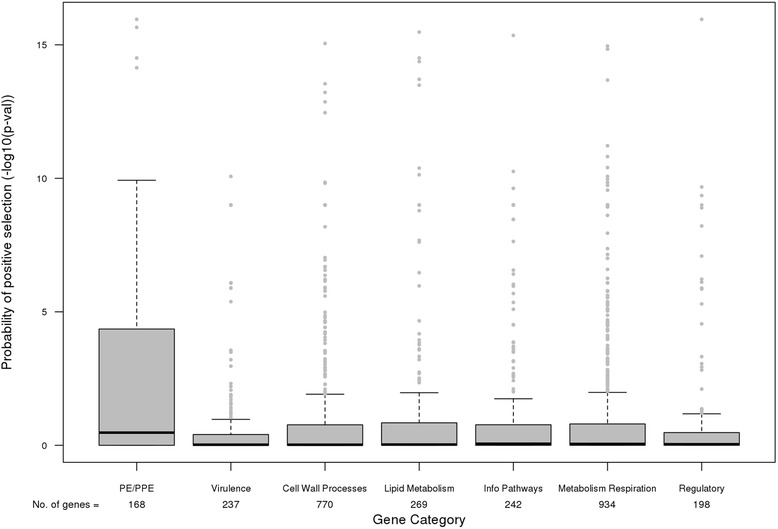
Evidence of positive selection between the *pe/ppe* and other genes by functional annotation. Distributions of (−log10) *p*-values for positive selection (evidence of ω >1) across the *pe/ppe* and other genes by functional annotation

In most genes it would be expected that only a small subset of sites would undergo positive selection and so calculation of a single *ω* value over all sites in the gene may dilute an effect. For example, this is possible in *pe/ppe* genes where there is less variation in the N- compared to the C- terminus [21]. We therefore used a likelihood ratio based approach that accounts for the variability of ω between sites. After implementation, we detected a greater proportion of *pe/ppe* loci under positive selection compared to other genes (*ω* > 1 and *P* < 0.05: *pe/ppe* genes 65 (39 %) vs. other genes 590 (15 %)). This observation remained consistent when the non-*pe/ppe* genes were subdivided into functional categories (*P*-values for evidence of *ω* >1, Wilcoxon *P* <0.001) (Fig. 4). Using the COG categories, the genes associated with cell motility and the *pe/ppe* genes again showed greater evidence of significant positive selection (Additional file 10: Figure S9). All genes annotated as possible recombination hotspots were identified as being under positive selection, except *Rv0182c.* To localize the specific polymorphisms under selection we applied the Bayes Empirical Bayes (BEB) method [29], and identified a small number of sites in each gene (median (range): *pe/ppe* genes 0 (0–60), other genes 0 (0–48), *P* = 1.2 × 10^−10^). In total 99 *pe/*ppe genes had sites under positive selection, including ten genes with selection at more than ten sites (Additional file 1: Table S5). For 1,106 non-*pe/*ppe genes, only 37 had ten or more sites under positive selection. The proportion of segregating sites under positive selection (*S*_*p*_*/S*_*s*_) per gene was higher in the *pe/ppe* loci compared to others (*pe/ppe* genes 0.04, other genes 0.00, Wilcoxon *P* = 2.58 × 10^−7^). There was a correlation between the number of positively selected and segregating sites (Pearson’s *r*, *pe/ppe* 0.81, and other genes 0.32).

We considered the 3,686 sites in the 1,106 non-*pe/ppe* genes with some evidence of positive selection (ω > 1). These sites were compared to a list of drug resistance-conferring mutations (www.tbdb.org), which because of a survival advantage may be expected to be under positive selection. Eighteen drug resistance markers were found, including in *inhA* (I21T, S94A, I194T, P251A; associated with the drug isoniazid), *katG* (S315T; isoniazid), *gyrA* (A90V; fluoroquinolones), *rpoB* (P45L, rifampicin), *rpoL* (K43R; rifampicin), and *ponA1* (P631S; rifampicin). Other regions of interest included *rodA* (T336S) involved in cell wall processes and required for survival in primary murine macrophages, and *pks6* (V504L) involved in lipid metabolism and in vitro growth. Repeating the recombination detection analysis after removing the sites under positive selection identified by the BEB method, revealed six genes that lost their statistical significance (*phiP* > 0.05, *eccA3, pe_pgrs4, pe_pgrs17, ppe59, Rv3466 and esxV*), leaving 10 as crossover hotspots (Fig. 3). Given that variation in these genes is not caused by positive selection it is highly likely that recombination hotspots are indeed present at these ten loci. The proportion of sites under selection was high for *lppA* (7 %) and *lppB* (43 %) loci. The BEB method identified 38 codons in *lppA*/B at which ω > 1, with almost all the related mutations present in lineage 2 (East-Asian) samples. None of these codons were in previously described conserved positions [30], implying that the core function of the protein was not disturbed, and the mutations may contribute to antigenic variation.

#### Selection on epitopes

Epitopes potentially binding to major histocompatibility complex molecules were predicted in all PE/PPE proteins using the netMHCpan software (Additional file 1: Table S6). The number of epitopes varied by *pe/ppe* gene (median 45, range 0 – 455). Some *pe/ppe* sites identified as being under selection using the BEB approach did overlap with regions predicted to be epitopes. In particular, for 10 genes (*pe6, pe_pgrs26, pe18, pe_pgrs49, pe_pgrs60, ppe27, ppe57, ppe59, ppe60 and ppe65*), more than 20 % of predicted epitopes had sites under positive selection (Additional file 1: Table S6).

## Discussion

Members of the PE/PPE family of proteins have been found to trigger innate immune responses, are targets of the adaptive immune system, and potentially a rich source of diagnostic and vaccine antigens. As large ‘omic studies in *M. tuberculosis* have often excluded *pe/ppe* genes from analysis (e.g. [3]), the understanding of their function and diversity is poor compared to other loci. Assessing diversity across *M. tuberculosis* strain types is critical, as lineages may vary in propensity to transmit and cause disease. By applying a *de novo* assembly approach, we were able to characterize accurately nearly all 168 *pe/ppe* genes in 518 isolates with high genomic coverage, representing lineages 1 (Indo-Oceanic), 2 (East-Asian), 3 (East-African-Indian) and 4 (Euro-American). After identifying ~50 k genome-wide SNPs from whole genome alignments, we confirmed that *pe/ppe* genes, especially the *pe_pgrs* family, have a high density of non-synonymous mutations compared to other *M. tuberculosis* loci. This observation is consistent with their involvement in antigenic variation and immune evasion, where proteins that are directly exposed to host immune surveillance tend to show higher levels of polymorphism. A lower degree of polymorphism in the *ppe* genes (compared to *pe_pgrs*) is likely to reflect a strong functional constraint of the PPE proteins.

Using all SNPs in a phylogenetic analysis, we observed clustering by *M. tuberculosis* lineage and therefore consistency with other published topologies [3, 31]. There was evidence of lineage specific *pe/ppe* repertories, with a very similar phylogeny being attained by restricting analysis to all polymorphisms in 167 PE/PPE genes (excluding *pe_pgrs3*), as well as a derived subset of 87 informative SNPs. The *pe_pgrs3* gene had high nucleotide diversity across all lineages, was not lineage informative, and is likely to be have been subject to recombination in lineages 1, 3 and 4. Both *M. bovis* and *M. canetti* contain two genes annotated as orthologues of *pe_pgrs3*, providing further evidence towards the propensity of this region to undergo genomic rearrangements. Interestingly the positioning of the *M. tuberculosis* reference strains in the *pe/ppe* gene phylogenetic tree was altered; some strains clustering near the *M. canetti* and ancestral strains while some of the known virulent reference strains were positioned at a further distance. Further study is needed to elucidate this effect. Other recombination and diversity hotspots included *lppB* (lineages 1, 2, and 4) and *ppe60* (lineage 4) genes*,* both known to have undergone homologous recombination. *LppB* (and *lppA)* are non-essential exported lipoproteins that are unique to pathogenic mycobacteria and may encode antigens [32, 33]. The *lppA/B* SNPs driving this effect were found mostly in lineage 2 (Beijing) strains, and seemed to be conferring a selective advantage. The role of lppA/B proteins on virulence should be investigated further. Although, *pe_pgrs17*, whose protein is in contact with the host immune system [34], was identified as a recombination hotspot, this observation may be confounded by positive selection. However, recombination has been described in *pe_pgrs17*, with large numbers of SNPs and indels in the *pe_pgrs17* and *pe_pgrs18* pair observed across the different lineages, potentially arising from gene conversion events [35]. We can rule out the results being confounded due to a sampling frame that included different geographical regions, as there was strongest clustering by lineage and not geographical source.

Across all *M. tuberculosis* genomes there was evidence that most genes were undergoing purifying selection pressures (*dN/dS* < 1). However, the *pe/ppe* genes were most likely to be under positive selection (*dN/dS* > 1), consistent with some PE/PPE proteins providing antigenic variation. It is possible the *dN/dS* ratios may be underestimated, as the methodology is more appropriate to divergent species and not for comparisons within a population [25]. Further, the signatures from very localised regions of selection within a gene may be diluted by surrounding genetic variation. A site-specific analysis confirmed the results from the gene-based *dN/dS*. Whilst the majority of the sixty-five genes identified as being under positive selection had only a single positively selected site, a disproportionate number of *pe_pgrs* genes had multiple positively selected sites. A potential limitation of this analysis is the time dependence of *dN/dS* for closely related bacterial genomes. This leads to possible over-estimation of the *dN/dS* and difficulties in interpretation when comparing the strength of selection between genes, genomes or populations over very short time-scales [36]. The power of the *dN/dS* statistic to detect positive selection is reduced when samples come from a single population [25]. In addition to the site under selection, multiple neighbouring and linked sites may show evidence of selection due to hitchhiking effects.

Our findings provide potential insights into the use of PE/PPE proteins as vaccine components. The high levels of polymorphism observed and the lineage-specific nature in certain members of these protein families could limit their effectiveness. A PE/PPE protein that displays higher sequence conservation across many strains may be a more effective vaccine candidate. For example, the highly immunogenic PE_PGRS62 protein has been considered as a vaccine target [37], and as only one of the 14 non-synonymous mutations observed was lineage specific, it may have broad strain coverage. However, one roadblock is the limited immunogenicity data available at the *pe/ppe* epitope level. It has been found that human T-cell epitopes are highly conserved in the *M. tuberculosis* complex [38], and like others [25] we found many epitopes predicted in PE/PPE proteins. Our analysis revealed a number of *pe/ppe* genes with a high proportion of epitopes potentially subjected to diversifying or positive selection. As these epitopes may be used by *M. tuberculosis* to evade the host immune system they would be relevant for TB vaccination strategies.

A cohesive understanding of the function of the 168 PE/PPE family of proteins remains elusive. By analysing SNP variation in 518 samples across the four main *M. tuberculosis* lineages we identified *pe/ppe* genes that are highly diverse, recombination hotspots and under positive selection. Such analyses can assist with prioritising candidates for functional studies, potentially leading to TB control measures, such as vaccines, diagnostics and drugs.

## Conclusions

Human tuberculosis poses a major burden on health services worldwide. There is a need to understand the complex interactions between the human host and bacterial pathogen so that new control measures, such as vaccines and drugs, can be developed. Recent technological advances have allowed large-scale studies to determine the genetic signatures of strain-types or ancestral lineages and drug resistance outcomes. Despite this advance, some highly variable regions of the genome are often excluded [39, 40]. This includes the *pe/ppe* gene family, whose members are thought to interact with the human immune system, but little is still known of their diversity and function. Here we present the first comprehensive study of the genetic diversity of the 168 *pe/ppe* genes. We find most genes vary in a lineage specific manner, consistent with strain-specific repertories. However, there were exceptions to this pattern, with evidence of some genes undergoing genetic cross-over events. Further, by looking for the genes under selective pressure genome-wide, we found enrichment in the number of *pe/ppe* genes undergoing positive selection. Overall, our work highlights the importance of *pe/ppe* genes, describes their suitability as vaccine candidates, and provides the basis for further exploration of the proteins involved in the host immune system and pathogen interactions.

## Methods

The raw sequencing fastq files for 518 *M. tuberculosis* samples with more than 100-fold genomic coverage were sourced from the PolyTB [41], rapid TB [42] and global drug resistance (Coll F, McNerney R, Hill-Cawthorn G et al. Whole genome association analysis of a global collection of Mycobacterium tuberculosis clinical isolates gives new insight into drug resistance, Submitted) projects (Additional file 1: Table S1a). A list of ENA accession numbers is available for download (http://pathogenseq.lshtm.ac.uk/ppe). Lineages were inferred using robust barcoding SNPs [3]. Lineages 1, 2, 3 and 4 were represented with 42, 38, 53 and 385 samples from each respectively (Additional file 1: Table S2). A separate set of twenty-one samples representing lineages 1 to 4 with complete or near complete genomes were used for validation (Additional file 1: Table S1b). In particular, all analyses performed on the main 518 samples were also applied to the validation dataset in an attempt to confirm signals and potentially rule out spurious findings. Assembly of all short reads was performed using MaSuRCA, SGA, Velvet and SPAdes [43–46] software, run in paired end mode with default and recommended parameters, across multiple k-mer values ranging from 31 to 91. The final Velvet run was implemented with a k-mer value of 63. Quast [47] software was used to extract assembly quality metrics using the H37Rv strain (Gene bank: AL123456) as the reference. The Samtools rmdup utility [48] was used to remove duplicates from each sample’s BAM file, and picard SamToFastq (http://broadinstitute.github.io/picard/) was used to convert the BAM files to fastq format. IMAGE software [49] was used to close gaps from the contigs produced by Velvet. After running IMAGE for 3 iterations using a k-mer size of 55, the number of *pe/ppe* genes assembled increased for all samples, especially in high coverage samples. The majority (range: 78–98 %) of gaps were closed within 3 iterations, which provided a threshold to justify the compromise between runtime and gaps closed in new contigs (fasta format). REAPR software was used to assess the quality of the assemblies, and calculates a quality score per base (http://www.sanger.ac.uk/science/tools/reapr reapr). The final assemblies are available for download (http://pathogenseq.lshtm.ac.uk/ppe). The *pe/ppe* and other genes were called by aligning the assemblies to the well annotated H37Rv genome. The 50,539 SNPs genome-wide were identified using nucmer [50] with H37Rv as the reference genome. To assess the robustness of the aligned sequences and resulting SNPs and analyses, we also mapped samples to a *Mycobacterium africanum* lineage reference (GCA_000253355.1), but observed no major differences from those using H37Rv (lineage 4). Phylogenetic data (alignments, phylogenetic trees) are deposited in Dryad (http://datadryad.org/).

The alignments of the genotypes for the 50,539 SNPs formed the basis of the majority of population genetic analyses, except where stated otherwise. SNP locations at which more than 10 % of the genotypes were missing were excluded from analyses. Other missing data was kept in the multiple alignments and was processed according to the default settings of the analysis software applied. Indels were identified by nucmer but were not analysed in this study. Regions where multiple contigs overlapped or where no contigs mapped to were annotated as missing data. FastTree [51] software employing the generalised time-reversible model was used to produce the final phylogenetic trees. The trees included the ancient *M. canettii* strain (NC_019950.1). The *F*_*ST*_ measure was calculated for each SNP to identify markers with complete between-lineage allele differentiation (*F*_*ST*_ >0.99). Similarly, the ancestral reconstructed sequence for the lineage-defining node in the phylogenetic tree was compared with its closest ancestral node, and the SNP differences derived. Nucleotide diversity (*π*) and the number of segregating sites were calculated using variscan software applied to sequence alignments [52]. To test for recombination we used the pairwise homoplasy index (*phi*) statistic calculated in sliding windows, as implemented in Phipack software [27]. The non-synonymous to synonymous ratio was calculated using PAML software [53]. To discover the effect of positive selection on the *pe/ppe* genes compared to all other genes, codeml was used to fit a number of models to the data using a maximum likelihood approach. This is generally thought to be more robust than counting methods. A *dN/dS (ω)* value was calculated per gene across all positions and all branches of the phylogenetic tree. For each gene, we then performed a likelihood ratio test using PAML software to assess evidence of positive selection, which compared two models: (a) variable selective pressure but no positive selection (0 < ω < 1) (M8a) and (b) variable selective pressure with positive selection (M8) (ω > 1). The test statistic has a *χ*2 (1 degree of freedom) distribution, and the resulting *p*-value reflects the likelihood of positive selection acting on a gene. To localize the specific polymorphisms under selection we applied the Bayes Empirical Bayes (BEB) method [29]. The proportion of segregating sites under positive selection (*S*_*p*_*/S*_*s*_) was calculated using the results from variscan and BEB. Epitopes were predicted using netMHCpan [54] using HLA alleles previously suggested [21].

No ethical approvals were required for this study.

### Availability of supporting data

The list of raw sequence data accession numbers for the ENA short read archive, final assemblies and links to the phylogenetic data (alignments, phylogenetic trees) in Dryad can be found in http://pathogenseq.lshtm.ac.uk/ppe.

## Additional files

Additional file 1: Table S1. a) The samples used for the assembly (*Malawi [55, 56], Netherlands [57], Pakistan [58], Portugal [59]) and b) the 21 reference strains. **Table S2.** Lineage, sequence coverage and polymorphism. *π* nucleotide diversity; Lineage 1 Indo-Oceanic; Lineage 2 East-Asian (Beijing); Lineage 3 East-African-Indian; Lineage 4 Euro-American. **Table S3.** Completeness of *pe/ppe* gene assemblies. **Table S4.** List of 87 *pe/ppe* lineage specific-markers. S synonymous, NS non-synonymous, * genes bolded if there are sites under selection using the Bayes Empirical Bayes method; Lineage 1 Indo-Oceanic; Lineage 2 East-Asian (Beijing); Lineage 3 East-African-Indian; Lineage 4 Euro-American. **Table S5.** Genes with more than 10 sites under selective pressure (*dN/dS (*ω) >1). **Table S6.** Epitopes. * identified using netMHCpan, ** epitopes that had sites under positive selection according to the Bayes Empirical Bayes (BEB) method. (DOCX 70 kb) Additional file 2: Figure S1. Allele frequency spectra for each lineage by synonymous (blue) and non-synonymous (red) mutations. The peaks at intermediate allele frequencies include sub-lineage defining SNPs (Lineage 1 Indo-Oceanic; Lineage 2 East-Asian (Beijing); Lineage 3 East-African-Indian; Lineage 4 Euro-American). (TIF 207 kb) Additional file 3: Figure S2. Gene-based nucleotide diversity (*π*) for the 21 reference genomes. All genes with high nucleotide diversity (*π* > 0.0075) are labelled. (TIF 148 kb) Additional file 4: Figure S3. Phylogenetic tree constructed using 50,540 genome-wide SNPs. Clear clustering according to lineage can be seen (Lineage 1 (Indo-Oceanic, green), lineage 2 (East-Asian (Beijing), blue), lineage 3 (East-African-Indian, purple), lineage 4 (Euro-American, red)). Reference genomes are labelled. *M. canetti* is annotated in cyan. (TIF 69 kb) Additional file 5: Figure S4. Identifying sites leading to differences in tree topologies based on all SNPs (Additional file 4: Figure S3a) and only those from *pe/ppe* genes (Additional file 4: Figure S3b). The Δ Site wise log likelihood score (Δ SSLS) is calculated for each SNP in the *pe/ppe* gene alignments. Negative differences indicate SNP positions favouring the *pe/ppe* tree. SNPs in *pe_pgrs3, ppe57* and *ppe60* produce strong phylogenetic signals supporting the *pe/ppe* tree. (TIF 113 kb) Additional file 6: Figure S5. Phylogenetic tree created using only SNPs from *pe_pgrs3*. No clear clustering by lineage is observed. However there are two major clades, one consistent with H37Rv (bottom-left). (TIF 126 kb) Additional file 7: Figure S6. Lineage-specific recombination hotspots. Manhattan plots showing genes that are likely to be recombination hotspots in each lineage (Lineage 1 Indo-Oceanic; Lineage 2 East-Asian (Beijing); Lineage 3 East-African-Indian; Lineage 4 Euro-American). The (−log10) p-value for the *phi* statistic is plotted against genome position. All genes with p-values < 0.05 are labelled. (TIF 147 kb) Additional file 8: Figure S7. Evidence of recombination at a gene level in the 21 reference genomes. A Manhattan plot showing genes that are likely to be recombination hotspots. The (−log10) p-value for the *phi* statistic is plotted against genome position. Genes with p-values less than 0.05 are shown. (TIF 120 kb) Additional file 9: Figure S8. Selection *dN/dS* values for each gene within Clusters of Orthologous Groups (COG*) categories. *ppe/N = *pe*/*ppe* genes annotated as COG category N, * COG categories: **A** RNA processing and modification, **B** Chromatin Structure and dynamics, **C** Energy production and conversion, **D** Cell cycle control and mitosis, **E** Amino Acid metabolism and transport, **F** Nucleotide metabolism and transport, **G** Carbohydrate metabolism and transport, **H** Coenzyme metabolism, **I** Lipid metabolism, **J** Translation, **K** Transcription, **L** Replication and repair, **M** Cell wall/membrane/envelope biogenesis, **N** Cell motility, **O** Post-translational modification, protein turnover, chaperone functions, **P** Inorganic ion transport and metabolism, **Q** Secondary Structure, **T** Signal Transduction, **U** Intracellular trafficking and secretion, **Y** Nuclear structure, **Z** Cytoskeleton, **R** General Functional Prediction only, **S** Function Unknown. (TIF 124 kb) Additional file 10: Figure S9. Non-neutral evolution for genes within Clusters of Orthologous Groups (COG*) categories. Boxplots are constructed using (-log10) p-values of non-neutral evolution for each gene. *ppe/N = *pe/ppe* genes annotated as COG category N, * COG categories: **A** RNA processing and modification, **B** Chromatin Structure and dynamics, **C** Energy production and conversion, **D** Cell cycle control and mitosis, **E** Amino Acid metabolism and transport, **F** Nucleotide metabolism and transport, **G** Carbohydrate metabolism and transport, **H** Coenzyme metabolism, **I** Lipid metabolism, **J** Translation, **K** Transcription, **L** Replication and repair, **M** Cell wall/membrane/envelope biogenesis, **N** Cell motility, **O** Post-translational modification, protein turnover, chaperone functions, **P** Inorganic ion transport and metabolism, **Q** Secondary Structure, **T** Signal Transduction, **U** Intracellular trafficking and secretion, **Y** Nuclear structure, **Z** Cytoskeleton, **R** General Functional Prediction only, **S** Function Unknown. (TIF 127 kb)
