# Supplementary material for: Recombination in pe/ppe genes contributes to genetic variation in Mycobacterium tuberculosis lineages
Source: BMC Genomics. 2016 Feb 29;17:151. doi: 10.1186/s12864-016-2467-y (PMC4770551; doi:10.1186/s12864-016-2467-y)
Supplement: Additional file 1: Table S1. — a) The samples used for the assembly (*Malawi [55, 56], Netherlands [57], Pakistan [58], Portugal [59]) and b) the 21 reference strains. Table S2. Lineage, sequence coverage and polymorphism. π nucleotide diversity; Lineage 1 Indo-Oceanic; Lineage 2 East-Asian (Beijing); Lineage 3 East-African-Indian; Lineage 4 Euro-American. Table S3. Completeness of pe/ppe gene assemblies. Table S4. List of 87 pe/ppe lineage specific-markers. S synonymous, NS non-synonymous, * genes bolded if there are sites under selection using the Bayes Empirical Bayes method; Lineage 1 Indo-Oceanic; Lineage 2 East-Asian (Beijing); Lineage 3 East-African-Indian; Lineage 4 Euro-American. Table S5. Genes with more than 10 sites under selective pressure (dN/dS (ω) >1). Table S6. Epitopes. * identified using netMHCpan, ** epitopes that had sites under positive selection according to the Bayes Empirical Bayes (BEB) method. (DOCX 70 kb) [file 12864_2016_2467_MOESM1_ESM.docx]

**S1 Table**

**a)**

| Study  location* | No.  samples | Lineage 1  Indo-Oceanic | Lineage 2  East-Asian | Lineage 3  East-African-Indian | Lineage 4  Euro-American |
| --- | --- | --- | --- | --- | --- |
| Brazil | 42 | - | - | - | 42 |
| Bulgaria | 2 | - | - | - | 2 |
| China | 6 | - | - | 5 | 1 |
| Malawi | 257 | 38 | 8 | 28 | 183 |
| Netherlands | 10 | - | - | - | 10 |
| Pakistan | 31 | 4 | 4 | 19 | 4 |
| Peru | 65 | - | 5 | - | 60 |
| Portugal | 78 | - | 5 | - | 73 |
| South Africa | 27 | - | 16 | 1 | 10 |
| **Total** | **518** | **42** | **38** | **53** | **385** |

*Malawi [56, 57], Netherlands [58], Pakistan [59], Portugal [60]

**b)**

| Strain | Assembly Accession | Lineage |
| --- | --- | --- |
| CDC1551 | GCA_000008585.1 | Lineage4 |
| CTRI_2 | GCA_000224435.1 | Lineage4 |
| F11 | GCA_000016925.1 | Lineage4 |
| 7199_99 | GCA_000331445.1 | Lineage4 |
| H37Ra | GCA_000016145.1 | Lineage4 |
| KZN_1435 | GCA_000023625.1 | Lineage4 |
| KZN_4207 | GCA_000154585.2 | Lineage4 |
| KZN_605 | GCA_000154605.2 | Lineage4 |
| RGTB327 | GCA_000277085.1 | Lineage4 |
| RGTB423 | GCA_000277105.1 | Lineage1 |
| Beijing_NITR203 | GCA_000364825.1 | Lineage2 |
| Erdman_ATCC_35801 | GCA_000350205.1 | Lineage4 |
| Haarlem | GCA_000153685.2 | Lineage4 |
| UT205 | GCA_000304555.1 | Lineage4 |
| W_148 | GCA_000193185.1 | Lineage2 |
| CAS_NITR204 | GCA_000389925.1 | Lineage3 |
| CCDC5079 | GCA_000270345.1 | Lineage2 |
| CCDC5180 | GCA_000270365.1 | Lineage2 |
| M.bovis_Pasteur_1173p2 | GCA_000009445.1 | Bovis |
| M323 | Genbank CP010873.1 | Lineage 2 |
| 18b | Genbank CP007299.1 | Lineage 2 |

**S2 Table**

| Lineage | *n*  (%) | Median Coverage across genome | Median Coverage across *pe/ppe*  genes | Median  *π*  across  genome | Median  *π*  across  *pe/ppe*  genes | No.  Lineage specific *pe/ppe* SNPs |
| --- | --- | --- | --- | --- | --- | --- |
| 1 | 42 (8.1) | 187.7 | 127.7 | 0.00009 | 0.00017 | 36 |
| 2 | 38 (7.3) | 319.7 | 151.1 | 0.00002 | 0.00007 | 15 |
| 3 | 53 (10.2) | 329.5 | 174.0 | 0.00004 | 0.00007 | 28 |
| 4 | 385 (74.3) | 268.3 | 150.9 | 0.00007 | 0.00016 | 8 |
| Overall | 518 | 283.5 | 155.4 | 0.00014 | 0.00027 | 87 |

**S3 Table**

| Locus | Gene | Length | Total length of gaps (prop. of gene length) | Proportion of samples fully assembled | No. SNPs | Non-synonymous SNPs |
| --- | --- | --- | --- | --- | --- | --- |
| *Rv0109* | *pe_pgrs1* | 1490 | 0 (0) | 0.99 | 23 | 13 |
| *Rv0124* | *pe_pgrs2* | 1463 | 0 (0) | 0.93 | 27 | 18 |
| *Rv0151c* | *pe1* | 1766 | 0 (0) | 0.99 | 32 | 20 |
| *Rv0152c* | *pe2* | 1577 | 0 (0) | 1 | 21 | 15 |
| *Rv0159c* | *pe3* | 1406 | 0 (0) | 1 | 18 | 13 |
| *Rv0160c* | *pe4* | 1508 | 0 (0) | 1 | 16 | 10 |
| *Rv0278c* | *pe_pgrs3* | 2873 | 0 (0) | 0.78 | 281 | 135 |
| *Rv0279c* | *pe_pgrs4* | 2513 | 241 (0.1) | 0.25 | 110 | 52 |
| *Rv0285* | *pe5* | 308 | 0 (0) | 1 | 4 | 2 |
| *Rv0297* | *pe_pgrs5* | 1775 | 0 (0) | 0.98 | 23 | 16 |
| *Rv0335c* | *pe6* | 515 | 0 (0) | 1 | 28 | 17 |
| *Rv0532* | *pe_pgrs6* | 1784 | 0 (0) | 0.95 | 69 | 46 |
| *Rv0578c* | *pe_pgrs7* | 3920 | 0 (0) | 0.75 | 120 | 55 |
| *Rv0742* | *pe_pgrs8* | 527 | 0 (0) | 0.99 | 3 | 2 |
| *Rv0746* | *pe_pgrs9* | 2351 | 23 (0.01) | 0.44 | 68 | 41 |
| *Rv0747* | *pe_pgrs10* | 2405 | 0 (0) | 0.56 | 188 | 100 |
| *Rv0754* | *pe_pgrs11* | 1754 | 0 (0) | 1 | 13 | 8 |
| *Rv0832* | *pe_pgrs12* | 413 | 0 (0) | 1 | 2 | 2 |
| *Rv0833* | *pe_pgrs13* | 2249 | 0 (0) | 0.77 | 63 | 42 |
| *Rv0834c* | *pe_pgrs14* | 2648 | 0 (0) | 0.92 | 62 | 26 |
| *Rv0872c* | *pe_pgrs15* | 1820 | 0 (0) | 1 | 16 | 9 |
| *Rv0916c* | *pe7* | 299 | 0 (0) | 1 | 3 | 3 |
| *Rv0977* | *pe_pgrs16* | 2771 | 0 (0) | 0.82 | 136 | 103 |
| *Rv0978c* | *pe_pgrs17* | 995 | 0 (0) | 0.51 | 33 | 19 |
| *Rv0980c* | *pe_pgrs18* | 1373 | 318 (0.23) | 0.14 | 48 | 26 |
| *Rv1040c* | *pe8* | 827 | 0 (0) | 1 | 4 | 3 |
| *Rv1067c* | *pe_pgrs19* | 2003 | 305.5 (0.15) | 0.12 | 81 | 40 |
| *Rv1068c* | *pe_pgrs20* | 1391 | 207 (0.15) | 0.2 | 5 | 5 |
| *Rv1087* | *pe_pgrs21* | 2303 | 0 (0) | 0.58 | 77 | 48 |
| *Rv1088* | *pe9* | 434 | 0 (0) | 1 | 3 | 2 |
| *Rv1089* | *pe10* | 362 | 0 (0) | 1 | 5 | 4 |
| *Rv1091* | *pe_pgrs22* | 2561 | 197 (0.08) | 0.13 | 55 | 28 |
| *Rv1172c* | *pe12* | 926 | 0 (0) | 1 | 6 | 3 |
| *Rv1195* | *pe13* | 299 | 0 (0) | 1 | 12 | 9 |
| *Rv1214c* | *pe14* | 332 | 0 (0) | 1 | 4 | 2 |
| *Rv1243c* | *pe_pgrs23* | 1688 | 0 (0) | 0.91 | 11 | 8 |
| *Rv1325c* | *pe_pgrs24* | 1811 | 0 (0) | 0.88 | 54 | 23 |
| *Rv1386* | *pe15* | 308 | 0 (0) | 1 | 3 | 2 |
| *Rv1396c* | *pe_pgrs25* | 1730 | 0 (0) | 0.96 | 36 | 20 |
| *Rv1430* | *pe16* | 1586 | 0 (0) | 1 | 12 | 10 |
| *Rv1441c* | *pe_pgrs26* | 1475 | 0 (0) | 0.85 | 14 | 10 |
| *Rv1450c* | *pe_pgrs27* | 3989 | 418 (0.1) | 0.3 | 55 | 29 |
| *Rv1452c* | *pe_pgrs28* | 2225 | 22 (0.01) | 0.49 | 51 | 19 |
| *Rv1468c* | *pe_pgrs29* | 1112 | 0 (0) | 1 | 16 | 7 |
| *Rv1646* | *pe17* | 932 | 0 (0) | 1 | 2 | 2 |
| *Rv1651c* | *pe_pgrs30* | 3035 | 0 (0) | 0.98 | 40 | 20 |
| *Rv1768* | *pe_pgrs31* | 1856 | 0 (0) | 0.97 | 22 | 17 |
| *Rv1788* | *pe18* | 299 | 0 (0) | 0.95 | 15 | 14 |
| *Rv1791* | *pe19* | 299 | 0 (0) | 0.99 | 17 | 13 |
| *Rv1803c* | *pe_pgrs32* | 1919 | 0 (0) | 1 | 27 | 17 |
| *Rv1806* | *pe20* | 299 | 0 (0) | 1 | 3 | 2 |
| *Rv1818c* | *pe_pgrs33* | 1496 | 0 (0) | 0.98 | 36 | 14 |
| *Rv1840c* | *pe_pgrs34* | 1547 | 0 (0) | 0.99 | 22 | 13 |
| *Rv1983* | *pe_pgrs35* | 1676 | 0 (0) | 1 | 14 | 9 |
| *Rv2098c* | *pe_pgrs36* | 1304 | 0 (0) | 0.99 | 7 | 5 |
| *Rv2099c* | *pe21* | 173 | 0 (0) | 1 | 3 | 2 |
| *Rv2107* | *pe22* | 296 | 0 (0) | 1 | 2 | 1 |
| *Rv2126c* | *pe_pgrs37* | 770 | 0 (0) | 0.99 | 21 | 12 |
| *Rv2162c* | *pe_pgrs38* | 1598 | 0 (0) | 0.79 | 45 | 16 |
| *Rv2328* | *pe23* | 1148 | 0 (0) | 1 | 9 | 7 |
| *Rv2340c* | *pe_pgrs39* | 1241 | 0 (0) | 1 | 16 | 9 |
| *Rv2371* | *pe_pgrs40* | 185 | 0 (0) | 1 | 1 | 0 |
| *Rv2396* | *pe_pgrs41* | 1085 | 0 (0) | 0.91 | 26 | 15 |
| *Rv2408* | *pe24* | 719 | 0 (0) | 1 | 5 | 4 |
| *Rv2431c* | *pe25* | 299 | 0 (0) | 1 | 3 | 2 |
| *Rv2487c* | *pe_pgrs42* | 2084 | 0 (0) | 0.85 | 21 | 10 |
| *Rv2490c* | *pe_pgrs43* | 4982 | 14 (0) | 0.43 | 103 | 44 |
| *Rv2519* | *pe26* | 1478 | 0 (0) | 1 | 19 | 11 |
| *Rv2591* | *pe_pgrs44* | 1631 | 0 (0) | 0.96 | 19 | 12 |
| *Rv2615c* | *pe_pgrs45* | 1385 | 0 (0) | 0.51 | 27 | 11 |
| *Rv2634c* | *pe_pgrs46* | 2336 | 0 (0) | 0.97 | 21 | 10 |
| *Rv2741* | *pe_pgrs47* | 1577 | 0 (0) | 0.86 | 56 | 33 |
| *Rv2769c* | *pe27* | 827 | 0 (0) | 1 | 13 | 11 |
| *Rv2853* | *pe_pgrs48* | 1847 | 0 (0) | 0.98 | 24 | 17 |
| *Rv3018A* | *pe27A* | 86 | 0 (0) | 0.69 | 0 | 0 |
| *Rv3022A* | *pe29* | 314 | 0 (0) | 0.98 | 0 | 0 |
| *Rv3344c* | *pe_pgrs49* | 1454 | 0 (0) | 0.78 | 47 | 16 |
| *Rv3345c* | *pe_pgrs50* | 4616 | 125 (0.03) | 0.22 | 207 | 105 |
| *Rv3367* | *pe_pgrs51* | 1766 | 0 (0) | 1 | 15 | 7 |
| *Rv3388* | *pe_pgrs52* | 2195 | 0 (0) | 0.76 | 48 | 33 |
| *Rv3477* | *pe31* | 296 | 0 (0) | 1 | 10 | 8 |
| *Rv3507* | *pe_pgrs53* | 4145 | 0 (0) | 0.64 | 133 | 97 |
| *Rv3508* | *pe_pgrs54* | 5705 | 2018 (0.35) | 0 | 358 | 200 |
| *Rv3511* | *pe_pgrs55* | 2144 | 233 (0.11) | 0.2 | 119 | 74 |
| *Rv3512* | *pe_pgrs56* | 3239 | 345 (0.11) | 0.04 | 174 | 114 |
| *Rv3514* | *pe_pgrs57* | 4469 | 2651 (0.59) | 0 | 39 | 29 |
| *Rv3590c* | *pe_pgrs58* | 1754 | 8 (0) | 0.48 | 49 | 17 |
| *Rv3595c* | *pe_pgrs59* | 1319 | 0 (0) | 1 | 22 | 8 |
| *Rv3622c* | *pe32* | 299 | 0 (0) | 0.99 | 3 | 2 |
| *Rv3650* | *pe33* | 284 | 0 (0) | 1 | 4 | 3 |
| *Rv3652* | *pe_pgrs60* | 314 | 0 (0) | 1 | 7 | 4 |
| *Rv3653* | *pe_pgrs61* | 587 | 0 (0) | 0.99 | 10 | 8 |
| *Rv3746c* | *pe34* | 335 | 0 (0) | 1 | 8 | 8 |
| *Rv3812* | *pe_pgrs62* | 1514 | 0 (0) | 1 | 20 | 14 |
| *Rv3872* | *pe35* | 299 | 0 (0) | 1 | 3 | 3 |
| *Rv3893c* | *pe36* | 233 | 0 (0) | 1 | 1 | 1 |
| *Rv0096* | *ppe1* | 1391 | 0 (0) | 1 | 27 | 19 |
| *Rv0256c* | *ppe2* | 1670 | 0 (0) | 1 | 17 | 9 |
| *Rv0280* | *ppe3* | 1610 | 0 (0) | 1 | 18 | 12 |
| *Rv0286* | *ppe4* | 1541 | 0 (0) | 1 | 16 | 11 |
| *Rv0304c* | *ppe5* | 6614 | 0 (0) | 0.97 | 65 | 37 |
| *Rv0305c* | *ppe6* | 2891 | 0 (0) | 1 | 36 | 22 |
| *Rv0354c* | *ppe7* | 425 | 0 (0) | 1 | 4 | 3 |
| *Rv0355c* | *ppe8* | 9902 | 12 (0) | 0.46 | 329 | 189 |
| *Rv0388c* | *ppe9* | 542 | 0 (0) | 1 | 11 | 2 |
| *Rv0442c* | *ppe10* | 1463 | 0 (0) | 0.98 | 14 | 9 |
| *Rv0453* | *ppe11* | 1556 | 0 (0) | 1 | 18 | 12 |
| *Rv0755c* | *ppe12* | 1937 | 0 (0) | 1 | 20 | 11 |
| *Rv0878c* | *ppe13* | 1331 | 0 (0) | 1 | 13 | 8 |
| *Rv0915c* | *ppe14* | 1271 | 0 (0) | 1 | 12 | 8 |
| *Rv1039c* | *ppe15* | 1175 | 0 (0) | 1 | 9 | 7 |
| *Rv1135c* | *ppe16* | 1856 | 0 (0) | 1 | 22 | 18 |
| *Rv1168c* | *ppe17* | 1040 | 0 (0) | 1 | 9 | 7 |
| *Rv1196* | *ppe18* | 1175 | 0 (0) | 0.56 | 6 | 2 |
| *Rv1361c* | *ppe19* | 1190 | 0 (0) | 0.7 | 53 | 31 |
| *Rv1387* | *ppe20* | 1619 | 0 (0) | 1 | 16 | 11 |
| *Rv1548c* | *ppe21* | 2036 | 0 (0) | 0.99 | 24 | 19 |
| *Rv1705c* | *ppe22* | 1157 | 0 (0) | 0.99 | 20 | 13 |
| *Rv1706c* | *ppe23* | 1184 | 0 (0) | 0.99 | 10 | 4 |
| *Rv1753c* | *ppe24* | 3161 | 282 (0.09) | 0 | 68 | 35 |
| *Rv1787* | *ppe25* | 1097 | 375 (0.34) | 0.36 | 6 | 5 |
| *Rv1789* | *ppe26* | 1181 | 0 (0) | 0.94 | 13 | 8 |
| *Rv1790* | *ppe27* | 1052 | 0 (0) | 0.53 | 12 | 10 |
| *Rv1800* | *ppe28* | 1967 | 0 (0) | 1 | 33 | 27 |
| *Rv1801* | *ppe29* | 1271 | 0 (0) | 0.99 | 9 | 4 |
| *Rv1802* | *ppe30* | 1391 | 0 (0) | 1 | 25 | 17 |
| *Rv1807* | *ppe31* | 1199 | 0 (0) | 1 | 12 | 6 |
| *Rv1808* | *ppe32* | 1229 | 0 (0) | 1 | 13 | 5 |
| *Rv1809* | *ppe33* | 1406 | 0 (0) | 1 | 14 | 9 |
| *Rv1917c* | *ppe34* | 4379 | 348 (0.08) | 0 | 132 | 63 |
| *Rv1918c* | *ppe35* | 2963 | 0 (0) | 0.98 | 54 | 34 |
| *Rv2108* | *ppe36* | 731 | 0 (0) | 1 | 10 | 7 |
| *Rv2123* | *ppe37* | 1421 | 0 (0) | 0.99 | 20 | 14 |
| *Rv2352c* | *ppe38* | 1175 | 0 (0) | 0.92 | 37 | 15 |
| *Rv2353c* | *ppe39* | 1064 | 0 (0) | 0.56 | 73 | 40 |
| *Rv2356c* | *ppe40* | 1847 | 0 (0) | 0.95 | 23 | 11 |
| *Rv2430c* | *ppe41* | 584 | 0 (0) | 0.92 | 6 | 3 |
| *Rv2608* | *ppe42* | 1742 | 0 (0) | 1 | 11 | 5 |
| *Rv2768c* | *ppe43* | 1184 | 0 (0) | 1 | 17 | 12 |
| *Rv2770c* | *ppe44* | 1148 | 0 (0) | 1 | 14 | 10 |
| *Rv2892c* | *ppe45* | 1226 | 0 (0) | 0.99 | 10 | 7 |
| *Rv3018c* | *ppe46* | 1304 | 151.5 (0.12) | 0.45 | 22 | 12 |
| *Rv3021c* | *ppe47* | 1076 | 223.5 (0.21) | 0.09 | 4 | 1 |
| *Rv3022c* | *ppe48* | 242 | 133.5 (0.55) | 0.36 | 1 | 0 |
| *Rv3125c* | *ppe49* | 1175 | 0 (0) | 0.98 | 26 | 17 |
| *Rv3135* | *ppe50* | 398 | 0 (0) | 0.68 | 0 | 0 |
| *Rv3136* | *ppe51* | 1142 | 0 (0) | 1 | 17 | 10 |
| *Rv3144c* | *ppe52* | 1229 | 0 (0) | 1 | 10 | 6 |
| *Rv3159c* | *ppe53* | 1772 | 0 (0) | 0.99 | 24 | 13 |
| *Rv3343c* | *ppe54* | 7571 | 543.5 (0.07) | 0.06 | 163 | 80 |
| *Rv3347c* | *ppe55* | 9473 | 0 (0) | 0.55 | 0 | 0 |
| *Rv3350c* | *ppe56* | 11150 | 0 (0) | 0.54 | 0 | 0 |
| *Rv3425* | *ppe57* | 530 | 2 (0) | 0.46 | 37 | 35 |
| *Rv3426* | *ppe58* | 698 | 697 (1) | 0.49 | 0 | 0 |
| *Rv3429* | *ppe59* | 536 | 0 (0) | 0.89 | 86 | 73 |
| *Rv3478* | *ppe60* | 1181 | 0 (0) | 0.92 | 155 | 110 |
| *Rv3532* | *ppe61* | 1220 | 0 (0) | 1 | 11 | 9 |
| *Rv3533c* | *ppe62* | 1748 | 0 (0) | 0.99 | 16 | 6 |
| *Rv3539* | *ppe63* | 1439 | 0 (0) | 1 | 13 | 9 |
| *Rv3558* | *ppe64* | 1658 | 0 (0) | 1 | 17 | 14 |
| *Rv3621c* | *ppe65* | 1241 | 0 (0) | 1 | 19 | 14 |
| *Rv3738c* | *ppe66* | 947 | 0 (0) | 0.9 | 0 | 0 |
| *Rv3739c* | *ppe67* | 233 | 0 (0) | 0.9 | 2 | 1 |
| *Rv3873* | *ppe68* | 1106 | 0 (0) | 1 | 13 | 7 |
| *Rv3892c* | *ppe69* | 1199 | 0 (0) | 1 | 12 | 6 |
| *Rv1169c* | *pe11* | 302 | 0 (0) | 1 | 2 | 2 |
| *Rv3020c* | *pe28* | 293 | 0 (0) | 0.73 | 0 | 0 |
| *Rv3097c* | *pe_pgrs63* | 1313 | 0 (0) | 1 | 12 | 6 |

**S4 Table**

| **Position** | **Mutation** | **Locus Tag** | | **Gene** | **NS/S** | **Lineage** |
| --- | --- | --- | --- | --- | --- | --- |
| 132646 | G/T | *Rv0109* | *pe_pgrs1* | | NS | 1 |
| 189948 | C/G | *Rv0160c* | *pe4* | | S | 1 |
| 308312 | G/A | *Rv0256c* | *ppe2* | | S | 1 |
| 339508 | C/T | *Rv0280* | *ppe3* | | S | 1 |
| 362007 | G/A | *Rv0297* | *pe_pgrs5* | | NS | 1 |
| 368948 | T/C | *Rv0304c* | *ppe5* | | NS | 1 |
| 372149 | G/A | *Rv0304c* | *ppe5* | | NS | 1 |
| 426768 | C/T | *Rv0355c* | *ppe8* | | NS | 1 |
| 434327 | A/G | *Rv0355c* | *ppe8* | | NS | 1 |
| 673066 | C/G | *Rv0578c* | *pe_ pgrs7* | | S | 1 |
| 673344 | A/T | *Rv0578c* | *pe_ pgrs7* | | S | 1 |
| 846996 | G/A | *Rv0754* | *pe_ pgrs11* | | NS | 1 |
| 928483 | C/T | *Rv0834c* | *pe_pgrs14* | | NS | 1 |
| 977196 | G/A | *Rv0878c* | *ppe13* | | S | 1 |
| 1188917 | G/A | *Rv1067c* | *pe_pgrs19* | | NS | 1 |
| 1656178 | C/T | *Rv1468c* | *pe_pgrs29* | | NS | 1 |
| 1863660 | C/T | *Rv1651c* | *pe_pgrs30* | | NS | 1 |
| 2045849 | C/T | *Rv1803c* | *pe_pgrs32* | | NS | 1 |
| 2165256 | T/G | *Rv1917c* | *ppe34* | | NS | 1 |
| 2423785 | C/T | *Rv2162c* | *pe_pgrs38* | | NS | 1 |
| 2803867 | G/C | *Rv2490c* | *pe_ pgrs43* | | S | 1 |
| 2961099 | G/A | *Rv2634c* | *pe_ pgrs46* | | NS | 1 |
| 3053973 | C/T | *Rv2741* | *pe_ pgrs47* | | S | 1 |
| 3080282 | C/A | *Rv2770c* | *ppe44* | | NS | 1 |
| 3929996 | G/T | *Rv3507* | *pe_*pgrs*53* | | NS | 1 |
| 3936696 | A/G | *Rv3508* | *pe_pgrs54* | | NS | 1 |
| 3942239 | C/A | *Rv3512* | *pe_pgrs56* | | S | 1 |
| 3944807 | T/C | *Rv3512* | *pe_pgrs56* | | S | 1 |
| 3970112 | C/T | *Rv3532* | *ppe61* | | NS | 1 |
| 3979151 | T/A | *Rv3539* | *ppe63* | | NS | 1 |
| 3998895 | G/A | *Rv3558* | *ppe64* | | NS | 1 |
| 4061113 | G/T | *Rv3621c* | *ppe65* | | S | 1 |
| 4093719 | G/A | *Rv3652* | *pe_pgrs60* | | NS | 1 |
| 4277032 | G/C | *Rv3812* | *pe_pgrs62* | | NS | 1 |
| 4351759 | G/C | *Rv3873* | *ppe68* | | NS | 1 |
| 4375318 | G/A | *Rv3892c* | *ppe69* | | NS | 1 |
| 424981 | G/A | *Rv0355c* | *ppe8* | | S | 2 |
| 1212432 | C/A | *Rv1087* | *pe_pgrs21* | | S | 2 |
| 1217065 | C/A | *Rv1091* | *pe_pgrs22* | | S | 2 |
| 1217157 | A/C | *Rv1091* | *pe_pgrs22* | | NS | 2 |
| 1218658 | G/C | *Rv1091* | *pe_pgrs22* | | S | 2 |
| 1299305 | G/A | *Rv1168c* | *ppe17* | | NS | 2 |
| 1357308 | T/G | *Rv1214c* | *pe14* | | S | 2 |
| 1606673 | G/T | *Rv1430* | *pe16* | | S | 2 |
| 2601760 | G/A | *Rv2328* | *pe23* | | NS | 2 |
| 2706663 | G/T | *Rv2408* | *pe24* | | NS | 2 |
| 2922846 | C/T | *Rv2591* | *pe_pgrs44* | | S | 2 |
| 2922848 | A/T | *Rv2591* | *pe_pgrs44* | | NS | 2 |
| 3895585 | C/T | *Rv3478* | *ppe60* | | NS | 2 |
| 4032218 | G/A | *Rv3590c* | *pe_pgrs58* | | NS | 2 |
| 4032625 | G/T | *Rv3590c* | *pe_pgrs58* | | S | 2 |
| 178205 | C/G | *Rv0151c* | *pe1* | | S | 3 |
| 178453 | C/G | *Rv0151c* | *pe1* | | S | 3 |
| 188317 | A/G | *Rv0159c* | *pe3* | | NS | 3 |
| 189850 | A/G | *Rv0160c* | *pe4* | | NS | 3 |
| 308661 | A/G | *Rv0256c* | *ppe2* | | NS | 3 |
| 350088 | C/A | *Rv0286* | *ppe4* | | NS | 3 |
| 367718 | G/T | *Rv0304c* | *ppe5* | | NS | 3 |
| 369886 | C/G | *Rv0304c* | *ppe5* | | S | 3 |
| 428921 | G/A | *Rv0355c* | *ppe8* | | NS | 3 |
| 432459 | C/T | *Rv0355c* | *ppe8* | | NS | 3 |
| 531775 | C/G | *Rv0442c* | *ppe10* | | S | 3 |
| 623163 | C/T | *Rv0532* | *pe_pgrs6* | | NS | 3 |
| 674702 | A/T | *Rv0578c* | *pe_pgrs7* | | S | 3 |
| 840847 | C/T | *Rv0747* | *pe_pgrs10* | | S | 3 |
| 1488428 | C/T | *Rv1325c* | *pe_pgrs24* | | NS | 3 |
| 1489142 | C/T | *Rv1325c* | *pe_pgrs24* | | NS | 3 |
| 1856617 | C/T | *Rv1646* | *pe17* | | NS | 3 |
| 1863584 | G/T | *Rv1651c* | *pe_pgrs30* | | NS | 3 |
| 2051345 | G/A | *Rv1809* | *ppe33* | | NS | 3 |
| 2382289 | G/T | *Rv2123* | *ppe37* | | NS | 3 |
| 2836773 | C/T | *Rv2519* | *pe26* | | NS | 3 |
| 2943675 | G/A | *Rv2615c* | *pe_pgrs45* | | S | 3 |
| 2960592 | C/T | *Rv2634c* | *pe_pgrs46* | | NS | 3 |
| 3738364 | G/A | *Rv3344c* | *ppe52* | | NS | 3 |
| 3738364 | G/A | *Rv3345c* | *pe_pgrs50* | | S | 3 |
| 3740181 | T/C | *Rv3345c* | *pe_pgrs50* | | NS | 3 |
| 3741240 | C/T | *Rv3345c* | *pe_pgrs50* | | NS | 3 |
| 4375452 | G/A | *Rv3892c* | *ppe69* | | NS | 3 |
| 428698 | T/C | *Rv0355c* | *ppe8* | | S | 4 |
| 1618978 | C/T | *Rv1441c* | *pe_pgrs26* | | NS | 4 |
| 1931718 | C/G | *Rv1705c* | *ppe22* | | S | 4 |
| 2050822 | C/G | *Rv1808* | *ppe32* | | NS | 4 |
| 2167926 | G/A | *Rv1918c* | *ppe35* | | NS | 4 |
| 3079877 | G/A | *Rv2770c* | *ppe44* | | NS | 4 |
| 3466919 | G/C | *Rv3097c* | *lipY* | | S | 4 |
| 3510120 | G/T | *Rv3144c* | *ppe52* | | NS | 4 |

**S5 Table**

| Name | Locus | Length | Function | No. sites |
| --- | --- | --- | --- | --- |
| *pe_pgrs3* | *Rv0278c* | 2873 | *pe/ppe* | 49 |
| *pe_pgrs54* | *Rv3508* | 5705 | *pe/ppe* | 39 |
| *Rv0668* | *Rv0668* | 3950 | information pathways | 34 |
| *pe_pgrs57* | *Rv3514* | 4469 | *pe/ppe* | 33 |
| *ppe54* | *Rv3343c* | 7571 | *pe/ppe* | 32 |
| *pe_pgrs56* | *Rv3512* | 3239 | *pe/ppe* | 29 |
| *ppe55* | *Rv3347c* | 9473 | *pe/ppe* | 29 |
| *ppe56* | *Rv3350c* | 11150 | *pe/ppe* | 26 |
| *pks12* | *Rv2048c* | 12455 | lipid metabolism | 25 |
| *pe_pgrs28* | *Rv1452c* | 2225 | *pe/ppe* | 23 |
| *Rv2850c* | *Rv2850c* | 1889 | metabolism & respiration | 21 |
| *Rv0075* | *Rv0075* | 1172 | metabolism & respiration | 20 |
| *lppA* | *Rv2543* | 659 | cell wall & cell processes | 20 |
| *lppB* | *Rv2544* | 662 | cell wall & cell processes | 19 |
| *pe_pgrs50* | *Rv3345c* | 4616 | *pe/ppe* | 18 |
| *ppe57* | *Rv3425* | 530 | *pe/ppe* | 18 |
| *Rv1453* | *Rv1453* | 1265 | regulatory proteins | 18 |
| *ppsA* | *Rv2931* | 5630 | lipid metabolism | 18 |
| *Rv1722* | *Rv1722* | 1484 | lipid metabolism | 17 |
| *ctpJ* | *Rv3743c* | 1982 | cell wall & cell processes | 17 |
| *pe_pgrs17* | *Rv0978c* | 995 | *pe/ppe* | 16 |
| *pe_pgrs18* | *Rv0980c* | 1373 | *pe/ppe* | 16 |
| *fadE1* | *Rv0131c* | 1343 | lipid metabolism | 16 |
| *Rv1729c* | *Rv1729c* | 938 | lipid metabolism | 16 |
| *pe_pgrs19* | *Rv1067c* | 2003 | *pe/ppe* | 15 |
| *pe_pgrs4* | *Rv0279c* | 2513 | *pe/ppe* | 15 |
| *pe_pgrs16* | *Rv0977* | 2771 | *pe/ppe* | 14 |
| *Rv2978c* | *Rv2978c* | 1379 | insertion sequences & phages | 14 |
| *pe_pgrs21* | *Rv1087* | 2303 | *pe/ppe* | 13 |
| *pe_pgrs9* | *Rv0746* | 2351 | *pe/ppe* | 13 |
| *ppe8* | *Rv0355c* | 9902 | *pe/ppe* | 13 |
| *Rv0080* | *Rv0080* | 458 | NA | 13 |
| *treY* | *Rv1563c* | 2297 | virulence, detoxification & adaptation | 13 |
| *Rv2827c* | *Rv2827c* | 887 | NA | 13 |
| *Rv2082* | *Rv2082* | 2165 | NA | 12 |
| *pe_pgrs10* | *Rv0747* | 2405 | *pe/ppe* | 11 |
| *ppe10* | *Rv0442c* | 1463 | *pe/ppe* | 11 |
| *Rv0893c* | *Rv0893c* | 977 | lipid metabolism | 11 |
| *Rv1254* | *Rv1254* | 1151 | metabolism & respiration | 11 |
| *Rv1776c* | *Rv1776c* | 560 | regulatory proteins | 11 |
| *acrA1* | *Rv3391* | 1952 | lipid metabolism | 11 |

**S6 Table**

| Gene | No. epitopes found* | No. (%) sites disturbed** |
| --- | --- | --- |
| ***pe_pgrs49*** | **2** | **2 (100)** |
| ***ppe59*** | **55** | **45 (81.8)** |
| ***ppe60*** | **95** | **61 (64.2)** |
| ***pe_pgrs60*** | **15** | **5 (33.3)** |
| ***pe18*** | **16** | **5 (31.3)** |
| ***pe_pgrs26*** | **43** | **12 (27.9)** |
| ***ppe57*** | **38** | **10 (26.3)** |
| ***pe6*** | **40** | **9 (22.5)** |
| ***ppe65*** | **85** | **19 (22.4)** |
| ***ppe27*** | **105** | **22 (21)** |
| ***pe_pgrs12*** | **35** | **7 (20)** |
| *pe25* | 17 | 3 (17.6) |
| *pe_pgrs7* | 26 | 4 (15.4) |
| *pe_pgrs20* | 29 | 4 (13.8) |
| *ppe54* | 373 | 44 (11.8) |
| *ppe46* | 125 | 14 (11.2) |
| *ppe19* | 101 | 11 (10.9) |
| *ppe47* | 83 | 9 (10.8) |
| *ppe22* | 95 | 10 (10.5) |
| *pe_pgrs10* | 40 | 4 (10) |
| *ppe52* | 61 | 6 (9.8) |
| *pe_pgrs13* | 11 | 1 (9.1) |
| *pe_pgrs3* | 67 | 6 (9) |
| *ppe13* | 84 | 7 (8.3) |
| *pe3* | 123 | 10 (8.1) |
| *ppe28* | 144 | 10 (6.9) |
| *pe_pgrs38* | 30 | 2 (6.7) |
| *ppe38* | 93 | 6 (6.5) |
| *pe_pgrs16* | 79 | 5 (6.3) |
| *ppe25* | 111 | 7 (6.3) |
| *pe_pgrs36* | 16 | 1 (6.3) |
| *ppe30* | 100 | 6 (6) |
| *pe19* | 18 | 1 (5.6) |
| *pe_pgrs18* | 39 | 2 (5.1) |
| *pe_pgrs31* | 42 | 2 (4.8) |
| *Ppe34* | 194 | 9 (4.6) |
| *Ppe24* | 182 | 8 (4.4) |
| *pe1* | 145 | 6 (4.1) |
| *pe_pgrs63* | 106 | 4 (3.8) |
| *ppe3* | 117 | 4 (3.4) |
| *ppe18* | 92 | 3 (3.3) |
| *pe_pgrs29* | 32 | 1 (3.1) |
| *pe_pgrs45* | 32 | 1 (3.1) |
| *pe_pgrs50* | 98 | 3 (3.1) |
| *pe17* | 73 | 2 (2.7) |
| *ppe53* | 75 | 2 (2.7) |
| *pe_pgrs41* | 38 | 1 (2.6) |
| *ppe68* | 79 | 2 (2.5) |
| *pe8* | 46 | 1 (2.2) |
| *ppe8* | 316 | 6 (1.9) |
| *ppe43* | 107 | 2 (1.9) |
| *ppe5* | 174 | 3 (1.7) |
| *ppe1* | 117 | 2 (1.7) |
| *ppe26* | 103 | 1 (1) |
| *ppe11* | 105 | 1 (1) |
| *ppe45* | 111 | 1 (0.9) |
| *pe16* | 127 | 1 (0.8) |
| *pe10* | 17 | 0 (0) |
| *pe11* | 21 | 0 (0) |
| *pe12* | 59 | 0 (0) |
| *pe13* | 20 | 0 (0) |
| *pe14* | 25 | 0 (0) |
| *pe15* | 10 | 0 (0) |
| *pe20* | 25 | 0 (0) |
| *pe2* | 106 | 0 (0) |
| *pe21* | 6 | 0 (0) |
| *pe22* | 28 | 0 (0) |
| *pe23* | 70 | 0 (0) |
| *pe24* | 40 | 0 (0) |
| *pe26* | 85 | 0 (0) |
| *pe27* | 49 | 0 (0) |
| *pe27A* | 2 | 0 (0) |
| *pe28* | 25 | 0 (0) |
| *pe29* | 10 | 0 (0) |
| *pe31* | 13 | 0 (0) |
| *pe32* | 16 | 0 (0) |
| *pe33* | 13 | 0 (0) |
| *pe34* | 22 | 0 (0) |
| *pe35* | 9 | 0 (0) |
| *pe36* | 7 | 0 (0) |
| *pe4* | 122 | 0 (0) |
| *pe5* | 8 | 0 (0) |
| *pe7* | 13 | 0 (0) |
| *pe9* | 25 | 0 (0) |
| *pe_pgrs11* | 113 | 0 (0) |
| *pe_pgrs1* | 34 | 0 (0) |
| *pe_pgrs14* | 48 | 0 (0) |
| *pe_pgrs15* | 23 | 0 (0) |
| *pe_pgrs17* | 31 | 0 (0) |
| *pe_pgrs19* | 36 | 0 (0) |
| *pe_pgrs21* | 35 | 0 (0) |
| *pe_pgrs22* | 37 | 0 (0) |
| *pe_pgrs2* | 31 | 0 (0) |
| *pe_pgrs23* | 36 | 0 (0) |
| *pe_pgrs24* | 36 | 0 (0) |
| *pe_pgrs25* | 23 | 0 (0) |
| *pe_pgrs27* | 26 | 0 (0) |
| *pe_pgrs28* | 25 | 0 (0) |
| *pe_pgrs30* | 111 | 0 (0) |
| *pe_pgrs32* | 37 | 0 (0) |
| *pe_pgrs33* | 33 | 0 (0) |
| *pe_pgrs34* | 32 | 0 (0) |
| *pe_pgrs35* | 98 | 0 (0) |
| *pe_pgrs37* | 3 | 0 (0) |
| *pe_pgrs39* | 48 | 0 (0) |
| *pe_pgrs40* | 12 | 0 (0) |
| *pe_pgrs42* | 30 | 0 (0) |
| *pe_pgrs43* | 42 | 0 (0) |
| *pe_pgrs4* | 38 | 0 (0) |
| *pe_pgrs44* | 33 | 0 (0) |
| *pe_pgrs46* | 30 | 0 (0) |
| *pe_pgrs47* | 38 | 0 (0) |
| *pe_pgrs48* | 17 | 0 (0) |
| *pe_pgrs51* | 30 | 0 (0) |
| *pe_pgrs52* | 25 | 0 (0) |
| *pe_pgrs5* | 25 | 0 (0) |
| *pe_pgrs53* | 27 | 0 (0) |
| *pe_pgrs54* | 28 | 0 (0) |
| *pe_pgrs55* | 32 | 0 (0) |
| *pe_pgrs56* | 0 | NA |
| *pe_pgrs57* | 30 | 0 (0) |
| *pe_pgrs58* | 28 | 0 (0) |
| *pe_pgrs59* | 31 | 0 (0) |
| *pe_pgrs61* | 5 | 0 (0) |
| *pe_pgrs62* | 137 | 0 (0) |
| *pe_pgrs6* | 41 | 0 (0) |
| *pe_pgrs8* | 30 | 0 (0) |
| *pe_pgrs9* | 41 | 0 (0) |
| *ppe10* | 85 | 0 (0) |
| *ppe12* | 71 | 0 (0) |
| *ppe14* | 100 | 0 (0) |
| *ppe15* | 115 | 0 (0) |
| *ppe16* | 76 | 0 (0) |
| *ppe17* | 93 | 0 (0) |
| *ppe20* | 140 | 0 (0) |
| *ppe2* | 131 | 0 (0) |
| *ppe21* | 71 | 0 (0) |
| *ppe23* | 96 | 0 (0) |
| *ppe29* | 91 | 0 (0) |
| *ppe31* | 98 | 0 (0) |
| *ppe32* | 94 | 0 (0) |
| *ppe33* | 83 | 0 (0) |
| *ppe35* | 161 | 0 (0) |
| *ppe36* | 49 | 0 (0) |
| *ppe37* | 137 | 0 (0) |
| *ppe39* | 22 | 0 (0) |
| *ppe40* | 66 | 0 (0) |
| *ppe41* | 49 | 0 (0) |
| *ppe4* | 153 | 0 (0) |
| *ppe42* | 116 | 0 (0) |
| *ppe44* | 96 | 0 (0) |
| *ppe48* | 29 | 0 (0) |
| *ppe49* | 105 | 0 (0) |
| *ppe50* | 45 | 0 (0) |
| *ppe51* | 80 | 0 (0) |
| *ppe55* | 374 | 0 (0) |
| *ppe56* | 455 | 0 (0) |
| *ppe58* | 46 | 0 (0) |
| *ppe6* | 161 | 0 (0) |
| *ppe61* | 93 | 0 (0) |
| *ppe62* | 60 | 0 (0) |
| *ppe63* | 134 | 0 (0) |
| *ppe64* | 63 | 0 (0) |
| *ppe66* | 79 | 0 (0) |
| *ppe67* | 18 | 0 (0) |
| *ppe69* | 63 | 0 (0) |
| *ppe7* | 18 | 0 (0) |
| *ppe9* | 45 | 0 (0) |
