# Supplementary figures and images for: Recombination in pe/ppe genes contributes to genetic variation in Mycobacterium tuberculosis lineages
Source: BMC Genomics. 2016 Feb 29;17:151. doi: 10.1186/s12864-016-2467-y (PMC4770551; doi:10.1186/s12864-016-2467-y)

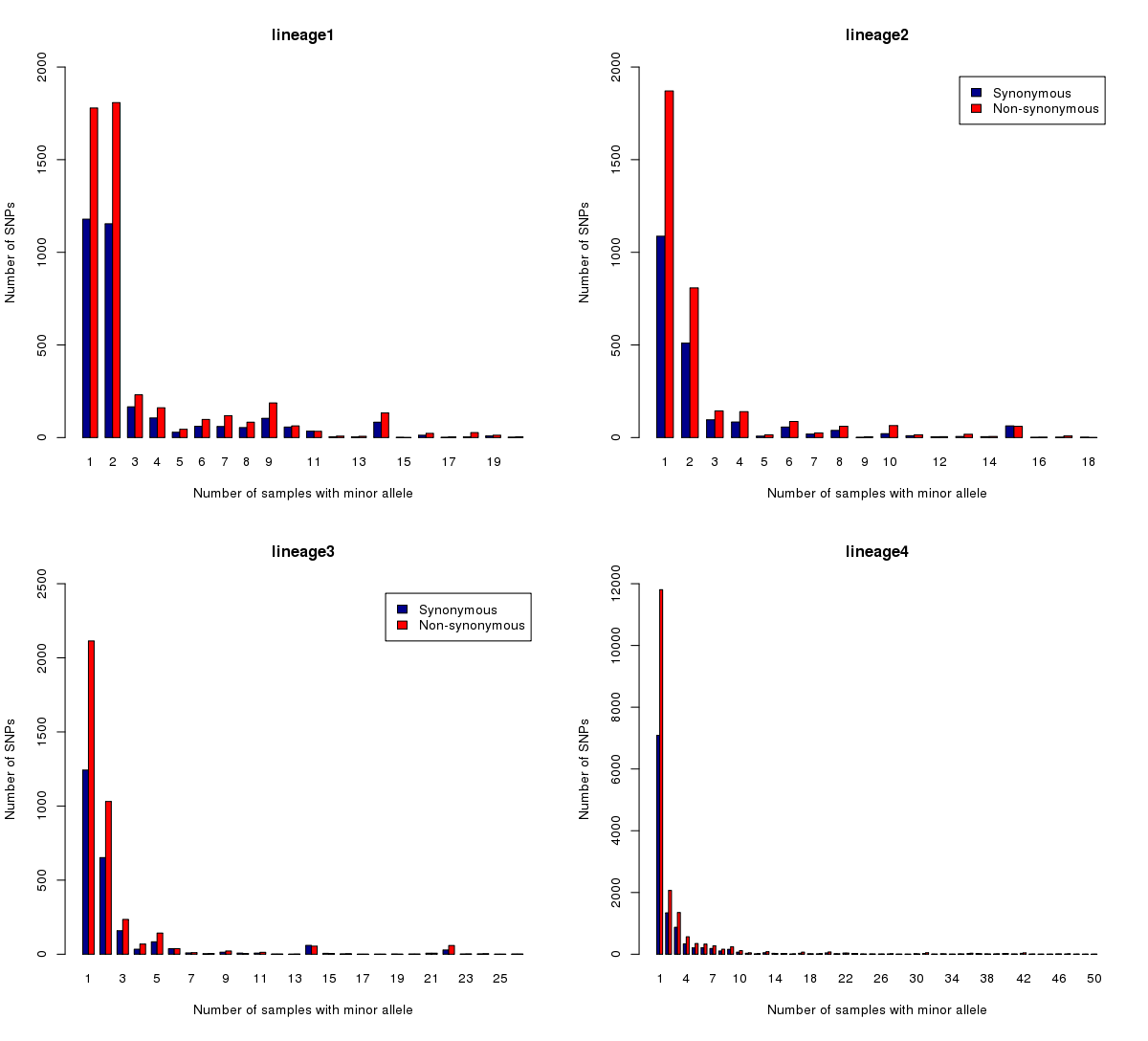

Supplement: Additional file 2: Figure S1. — Allele frequency spectra for each lineage by synonymous (blue) and non-synonymous (red) mutations. The peaks at intermediate allele frequencies include sub-lineage defining SNPs (Lineage 1 Indo-Oceanic; Lineage 2 East-Asian (Beijing); Lineage 3 East-African-Indian; Lineage 4 Euro-American). (TIF 207 kb) [file 12864_2016_2467_MOESM2_ESM.tif]

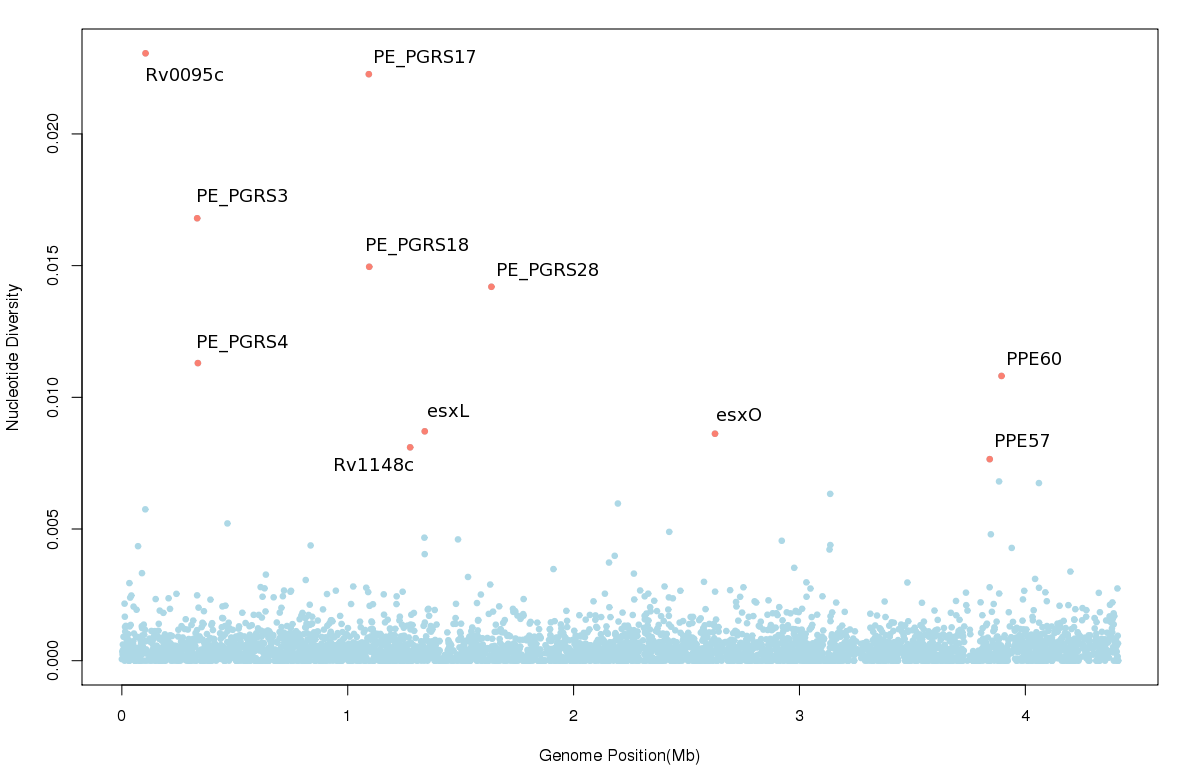

Supplement: Additional file 3: Figure S2. — Gene-based nucleotide diversity (π) for the 21 reference genomes. All genes with high nucleotide diversity (π > 0.0075) are labelled. (TIF 148 kb) [file 12864_2016_2467_MOESM3_ESM.tif]

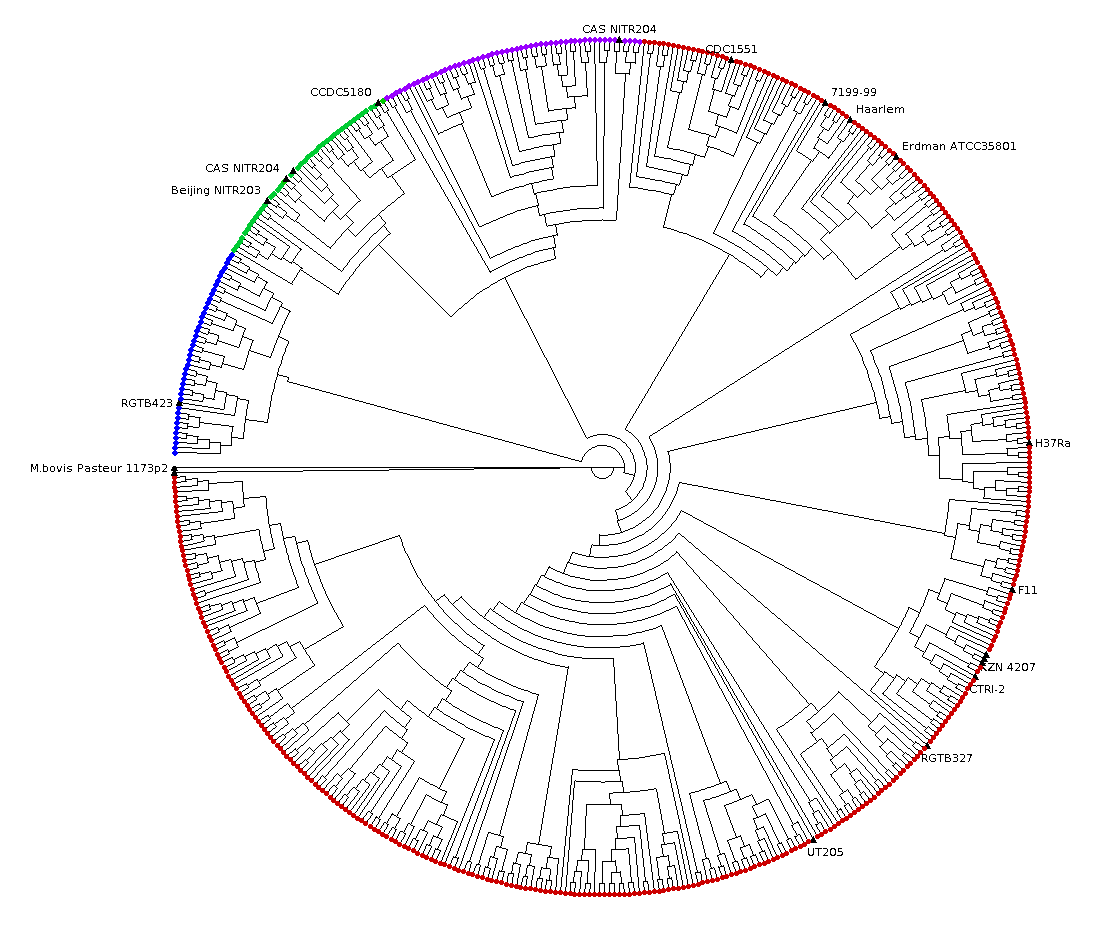

Supplement: Additional file 4: Figure S3. — Phylogenetic tree constructed using 50,540 genome-wide SNPs. Clear clustering according to lineage can be seen (Lineage 1 (Indo-Oceanic, green), lineage 2 (East-Asian (Beijing), blue), lineage 3 (East-African-Indian, purple), lineage 4 (Euro-American, red)). Reference genomes are labelled. M. canetti is annotated in cyan. (TIF 69 kb) [file 12864_2016_2467_MOESM4_ESM.tif]

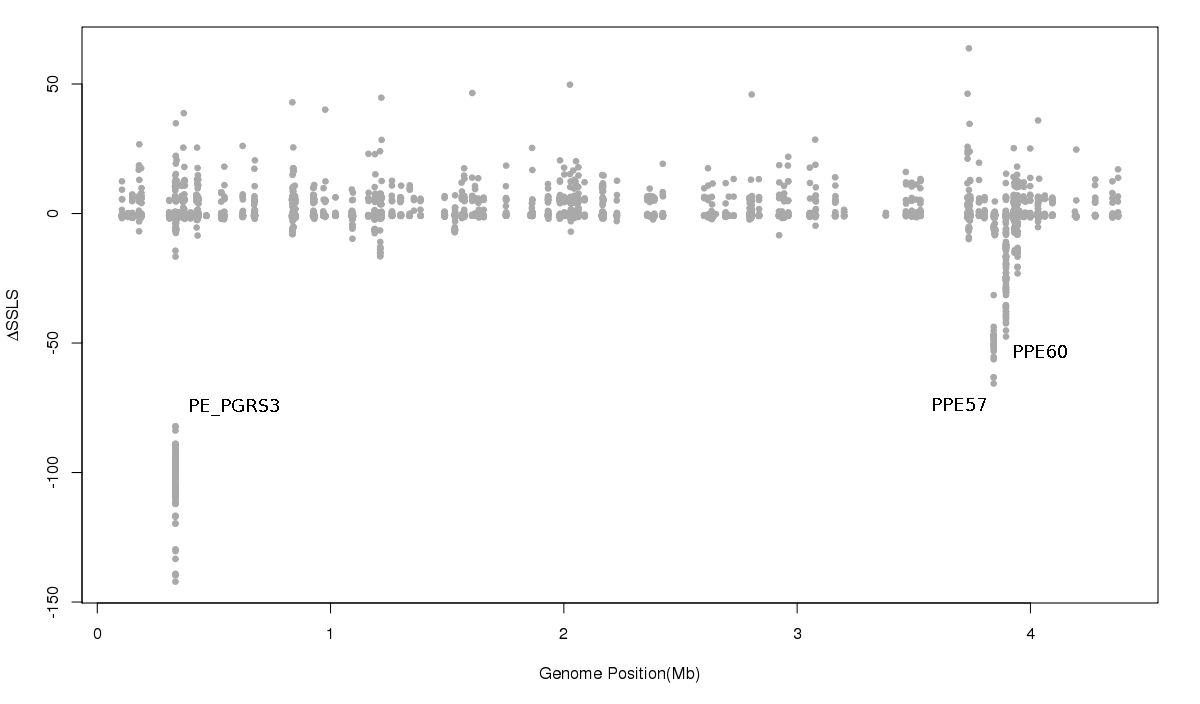

Supplement: Additional file 5: Figure S4. — Identifying sites leading to differences in tree topologies based on all SNPs (Additional file 4: Figure S3a) and only those from pe/ppe genes (Additional file 4: Figure S3b). The Δ Site wise log likelihood score (Δ SSLS) is calculated for each SNP in the pe/ppe gene alignments. Negative differences indicate SNP positions favouring the pe/ppe tree. SNPs in pe_pgrs3, ppe57 and ppe60 produce strong phylogenetic signals supporting the pe/ppe tree. (TIF 113 kb) [file 12864_2016_2467_MOESM5_ESM.tif]

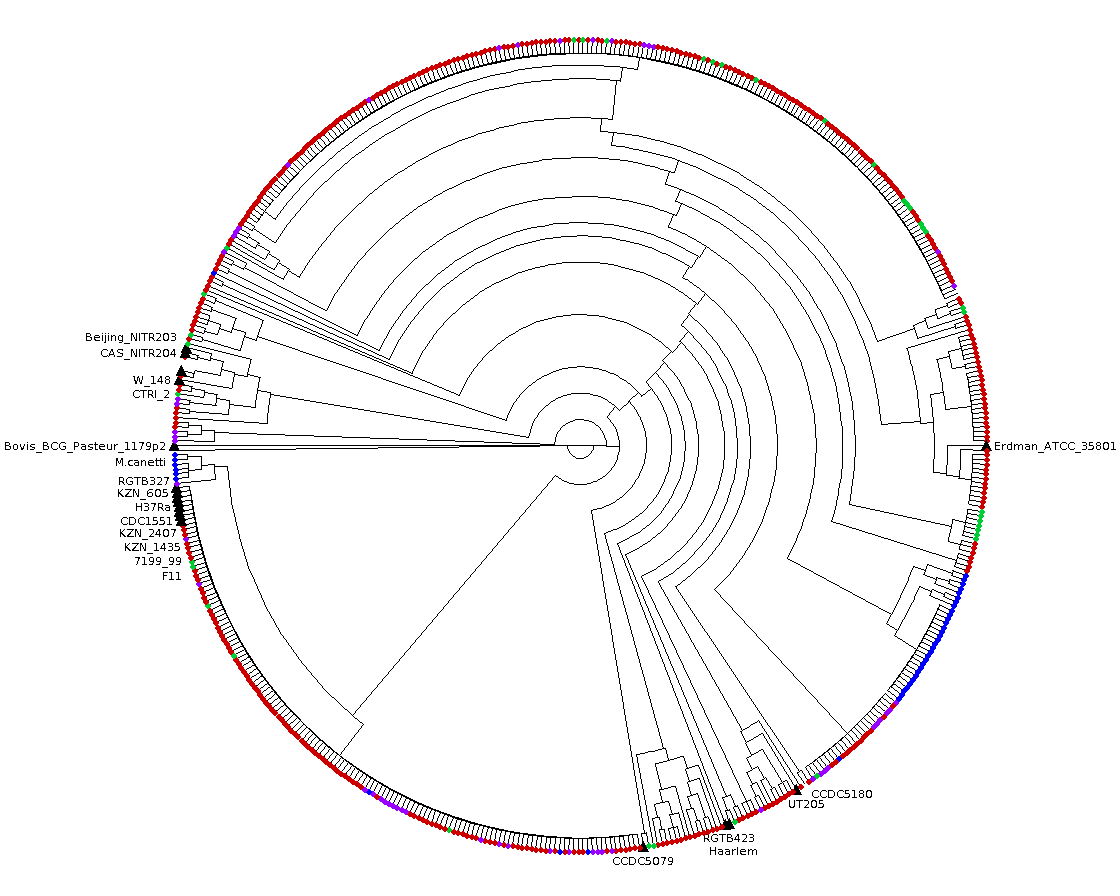

Supplement: Additional file 6: Figure S5. — Phylogenetic tree created using only SNPs from pe_pgrs3. No clear clustering by lineage is observed. However there are two major clades, one consistent with H37Rv (bottom-left). (TIF 126 kb) [file 12864_2016_2467_MOESM6_ESM.tif]

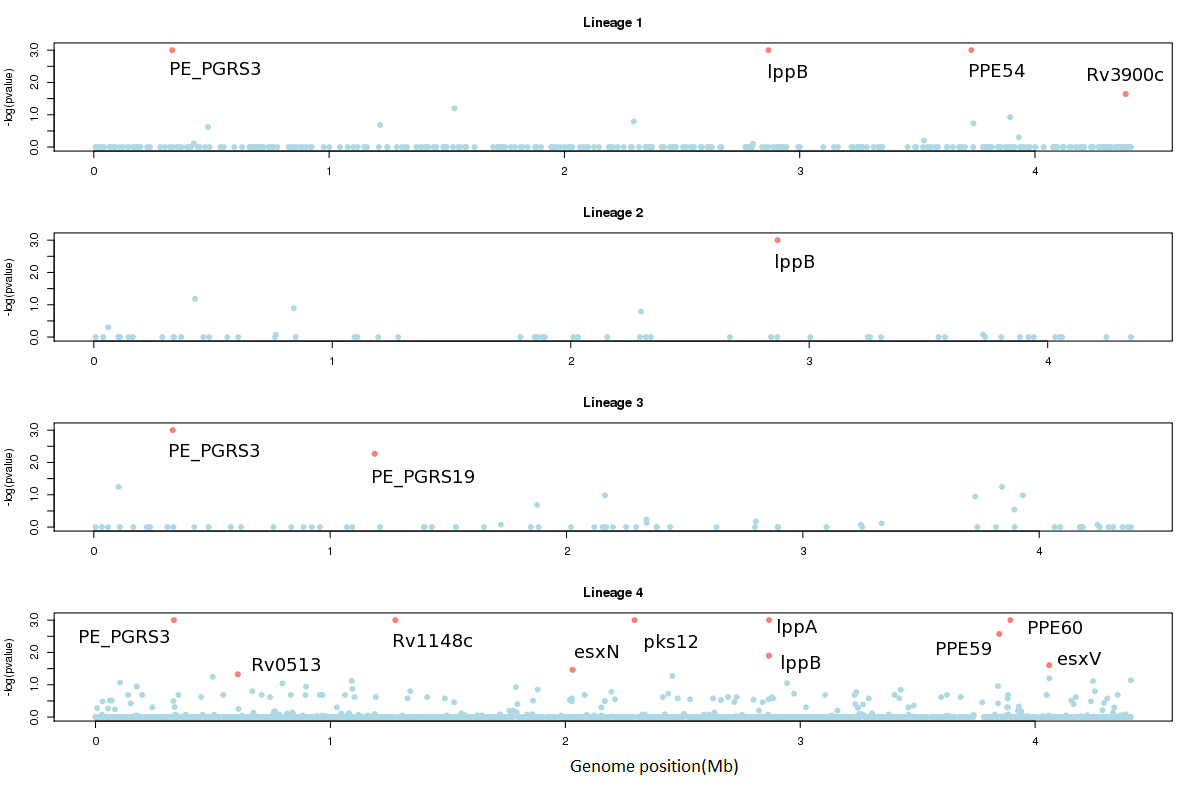

Supplement: Additional file 7: Figure S6. — Lineage-specific recombination hotspots. Manhattan plots showing genes that are likely to be recombination hotspots in each lineage (Lineage 1 Indo-Oceanic; Lineage 2 East-Asian (Beijing); Lineage 3 East-African-Indian; Lineage 4 Euro-American). The (−log10) p-value for the phi statistic is plotted against genome position. All genes with p-values < 0.05 are labelled. (TIF 147 kb) [file 12864_2016_2467_MOESM7_ESM.tif]

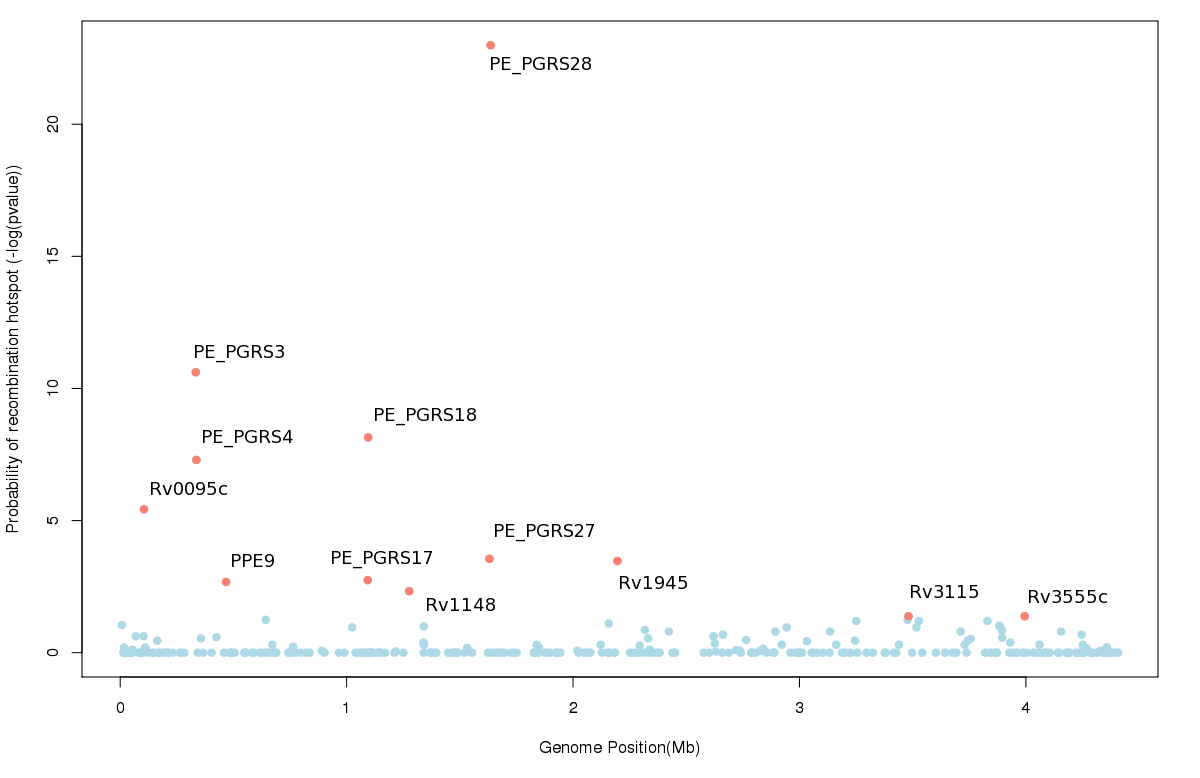

Supplement: Additional file 8: Figure S7. — Evidence of recombination at a gene level in the 21 reference genomes. A Manhattan plot showing genes that are likely to be recombination hotspots. The (−log10) p-value for the phi statistic is plotted against genome position. Genes with p-values less than 0.05 are shown. (TIF 120 kb) [file 12864_2016_2467_MOESM8_ESM.tif]

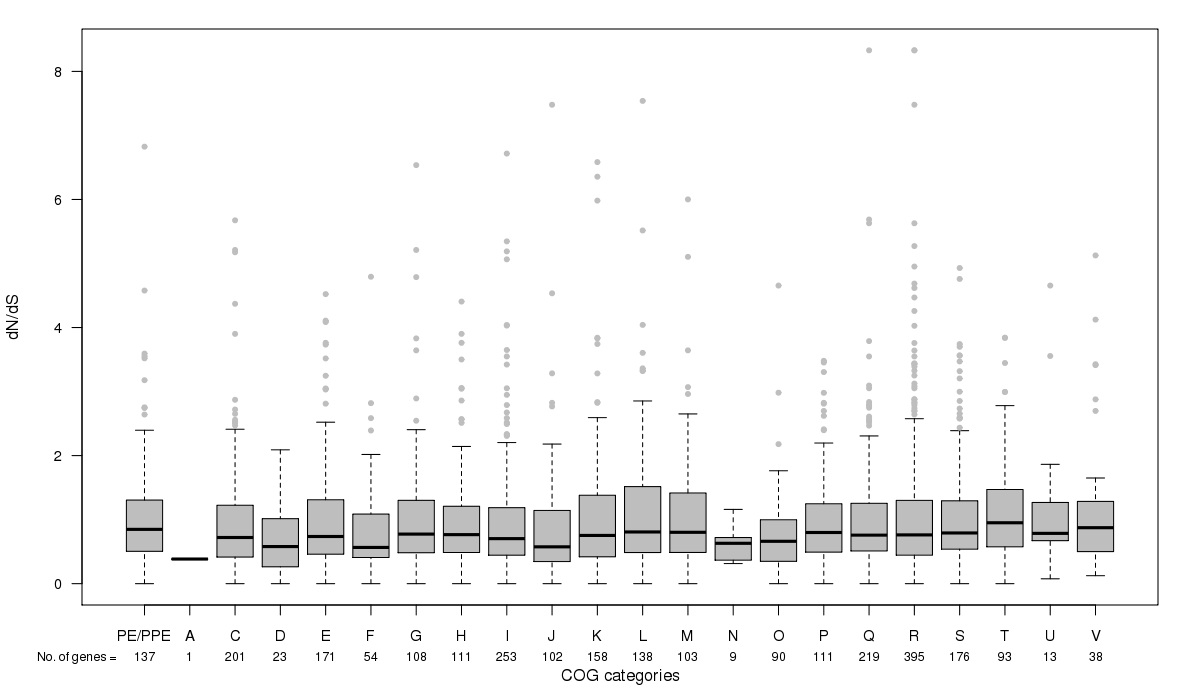

Supplement: Additional file 9: Figure S8. — Selection dN/dS values for each gene within Clusters of Orthologous Groups (COG*) categories. *ppe/N = pe/ppe genes annotated as COG category N, * COG categories: A RNA processing and modification, B Chromatin Structure and dynamics, C Energy production and conversion, D Cell cycle control and mitosis, E Amino Acid metabolism and transport, F Nucleotide metabolism and transport, G Carbohydrate metabolism and transport, H Coenzyme metabolism, I Lipid metabolism, J Translation, K Transcription, L Replication and repair, M Cell wall/membrane/envelope biogenesis, N Cell motility, O Post-translational modification, protein turnover, chaperone functions, P Inorganic ion transport and metabolism, Q Secondary Structure, T Signal Transduction, U Intracellular trafficking and secretion, Y Nuclear structure, Z Cytoskeleton, R General Functional Prediction only, S Function Unknown. (TIF 124 kb) [file 12864_2016_2467_MOESM9_ESM.tif]

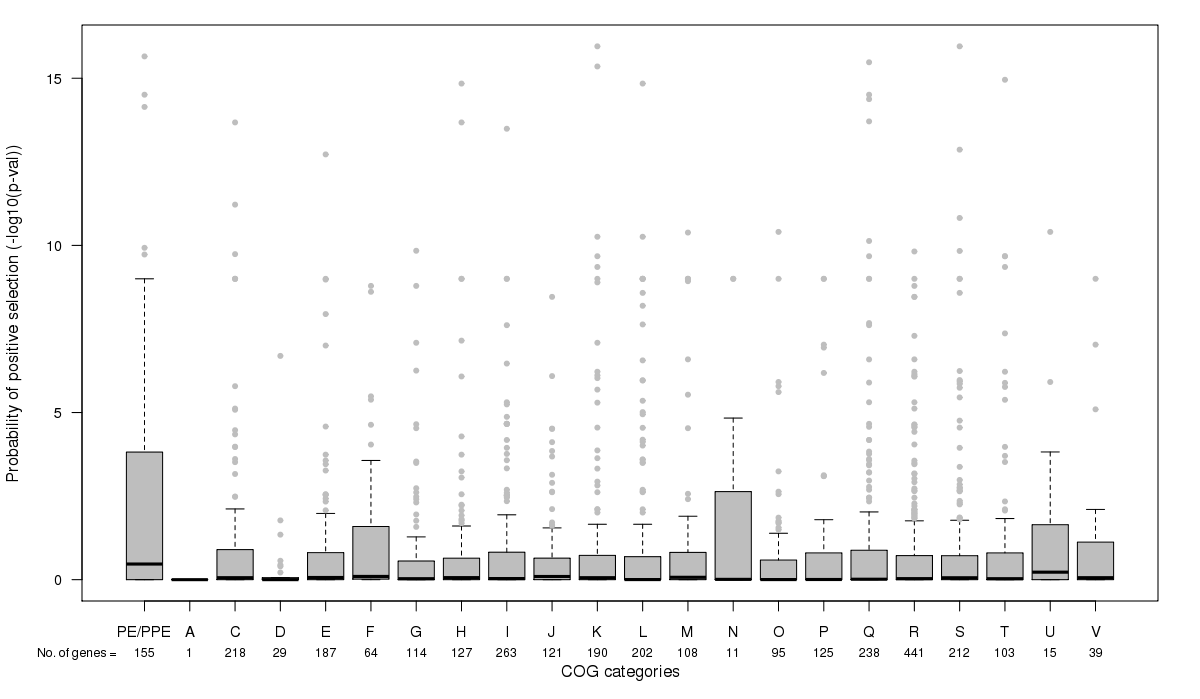

Supplement: Additional file 10: Figure S9. — Non-neutral evolution for genes within Clusters of Orthologous Groups (COG*) categories. Boxplots are constructed using (-log10) p-values of non-neutral evolution for each gene. *ppe/N = pe/ppe genes annotated as COG category N, * COG categories: A RNA processing and modification, B Chromatin Structure and dynamics, C Energy production and conversion, D Cell cycle control and mitosis, E Amino Acid metabolism and transport, F Nucleotide metabolism and transport, G Carbohydrate metabolism and transport, H Coenzyme metabolism, I Lipid metabolism, J Translation, K Transcription, L Replication and repair, M Cell wall/membrane/envelope biogenesis, N Cell motility, O Post-translational modification, protein turnover, chaperone functions, P Inorganic ion transport and metabolism, Q Secondary Structure, T Signal Transduction, U Intracellular trafficking and secretion, Y Nuclear structure, Z Cytoskeleton, R General Functional Prediction only, S Function Unknown. (TIF 127 kb) [file 12864_2016_2467_MOESM10_ESM.tif]
